# Single-cell transcriptomics reveals multi-step adaptations to endocrine therapy

**DOI:** 10.1038/s41467-019-11721-9

**Published:** 2019-09-02

**Authors:** Sung Pil Hong, Thalia E. Chan, Ylenia Lombardo, Giacomo Corleone, Nicole Rotmensz, Sara Bravaccini, Andrea Rocca, Giancarlo Pruneri, Kirsten R. McEwen, R. Charles Coombes, Iros Barozzi, Luca Magnani

**Affiliations:** 1 Department of Surgery and Cancer, Imperial College London, London, UK; 2 Department of Internal Medicine, Yonsei University College of Medicine, Seoul, Korea; 3 Centre for Integrative Systems Biology and Bioinformatics, Department of Life Sciences, Imperial College London, London, UK; 4 Division of Pathology, European Institute of Oncology and University of Milan, School of Medicine, Milan, Italy; 5 Istituto Scientifico Romagnolo per lo Studio e la Cura dei Tumori (IRST) IRCCS, Meldola, Italy; 6 Present Address: Nature Communications, The Macmillan Campus, 4 Crinan Street, London, N1 9XW UK

**Keywords:** Mechanisms of disease, Computational models, Breast cancer, Cancer therapeutic resistance

## Abstract

Resistant tumours are thought to arise from the action of Darwinian selection on genetically heterogenous cancer cell populations. However, simple clonal selection is inadequate to describe the late relapses often characterising luminal breast cancers treated with endocrine therapy (ET), suggesting a more complex interplay between genetic and non-genetic factors. Here, we dissect the contributions of clonal genetic diversity and transcriptional plasticity during the early and late phases of ET at single-cell resolution. Using single-cell RNA-sequencing and imaging we disentangle the transcriptional variability of plastic cells and define a rare subpopulation of pre-adapted (PA) cells which undergoes further transcriptomic reprogramming and copy number changes to acquire full resistance. We find evidence for sub-clonal expression of a PA signature in primary tumours and for dominant expression in clustered circulating tumour cells. We propose a multi-step model for ET resistance development and advocate the use of stage-specific biomarkers.

## Abstract (web summary)

The development of resistance to endocrine therapy is a significant, clinical problem in breast cancer. Here, the authors identify a rare subpopulation of cells that drive resistance following transcriptional reprogramming.

## Introduction

The outgrowth of primary luminal breast cancer (BCa) is driven by non-mutated oestrogen receptor α (ERα), with all patients receiving adjuvant endocrine therapy (ET) after curative surgery. This strategy significantly delays clinical relapse but does not abrogate it completely, as about ~3% of the patients each year come back with overt relapse, inevitably leading to further metastatic development^1–3^. The frequency of relapse remains constant up to 20 years after surgery making ET resistance the most critical clinical problem for the management of these patients^4^. The processes of adaptation and selection leading to late relapse are currently poorly understood, and should be interpreted in light of adjuvant therapies.

Recent developments in next-generation sequencing (NGS) revealed that tumours are genetically heterogeneous^5–7^, and in some cancer types, heterogeneity correlates with the likelihood of recurrence and development of drug resistance^8,9^. In some instances, targeted therapy can lead to the rapid expansion of genetically defined pre-existent resistant cells that can be explained by simple models of clonal selection^10–12^. However, this same model is mostly inconsistent with the decade-long latency observed in luminal BCa. In addition, despite recent studies showed that the majority of the genetic lesions in BCa are accumulated during the early phases of tumour development^5,13^, they failed to identify any major driver associated to metastasis and resistance, with the exception of a minor fraction of cases showing either *ESR1* mutations or *CYP19A1* amplification^14–17^. Yet, the transcriptomes of the resistant cells are profoundly heterogeneous and different from those of the primary tumour^18–20^, suggesting a contribution of non-genetic mechanisms^21^.

Rare phenotypic subpopulations, showing features of drug tolerance and sometimes of quiescence, have been found in primary melanomas^22^, leukaemia^23^, non-small-cell lung cancer^24^ and triple-negative breast cancer (TNBC)^25^. In primary melanoma, a rare, transient subpopulation expressing resistant markers at high levels can survive and persist to become stably resistant^26^. Nevertheless, it remains unclear how genetic and non-genetic components contribute to different types or stages of ERα-positive BCa.

In this study, we use a combination of live cell imaging, single-cell RNA-sequencing (scRNA-seq) and machine learning to dissect the phenotypic heterogeneity and plasticity of ERα-positive BCa, and leverage this information to identify a subpopulation of rare, pre-adapted cells both in vitro and in vivo. These cells (termed PA, from Pre-Adapted) display a unique transcriptional signature with features of dormancy and mixed epithelial and mesenchymal traits, which is found dominant in clusters of circulating tumour cells. PA cells show a significant survival advantage under short-term ET, but require further transcriptional reprogramming and genetic alterations to acquire full resistance and re-establish a proliferative phenotype in vitro. These results highlight the multi-faceted effects of ET at single-cell level, and suggest a multi-step mechanism of drug resistance that involve both non-genetic and genetic contributions.

## Results

### Absence of features of resistance in treatment-naive cells

**Fig. 1:**
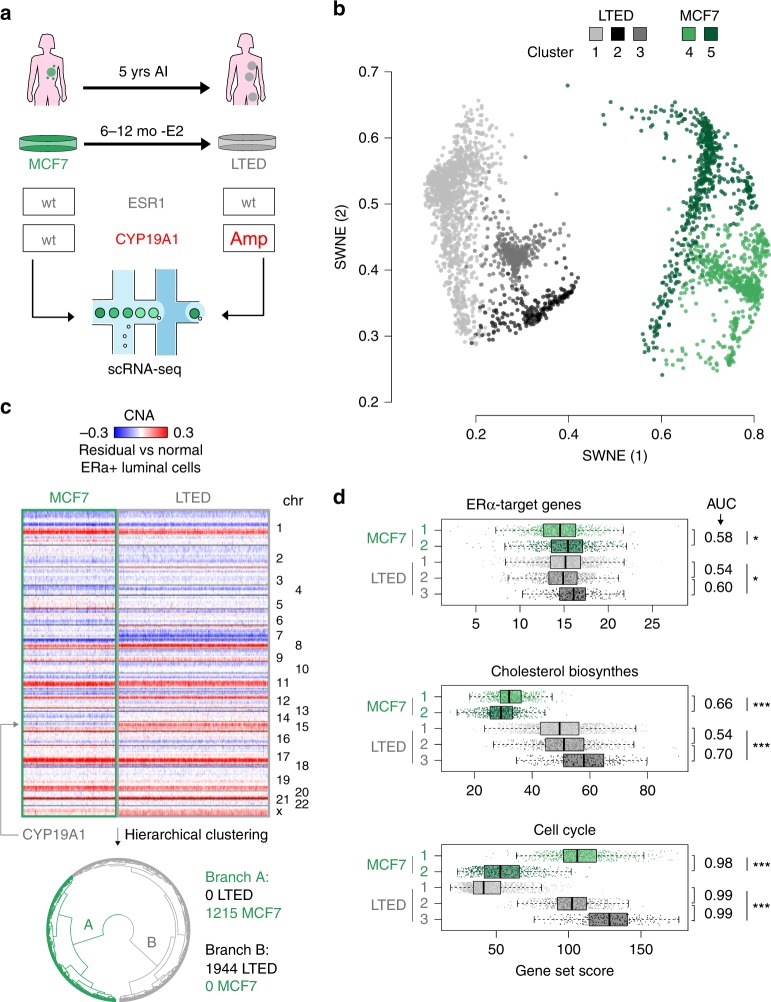
Absence of fully resistant clones in treatment-naive cells. **a** Schematic representation of the in vitro approach (bottom), which mimics the development of resistance to aromatase inhibitors (AI) in patients. **b** Bi-dimensional representation of 3159 single-cell transcriptomes (1125 MCF7 and 1944 LTED) (SWNE; *k* = 16). **c** Copy number profiles of the cells shown in (**b**), as estimated from scRNA-seq profiles. The data shown as heatmap and as dendrogram (hierarchical clustering; Ward’s method; Euclidean distance). **d** Distribution of normalised expression levels for selected gene sets, by cluster of cells (as defined in **b**). Area Under the Curve calculated using the cluster with higher median gene expression as a positive set. Box plots show median, interquartile values, range and outliers (individual points). *p <= 1e-5, **p <= 1e-10, *** *p* <= 2.2e-16 (Kruskal–Wallis test)

In order to study the dynamic process of ET resistance, we exploited an in vitro system that maximises reproducibility while minimising confounding factors^15,27^. Long-term oestrogen-deprived (LTED) cells originate from ESR1 wild-type MCF7 that have been deprived from oestradiol (E2) for 1 year. This model is generally considered a good proxy to study the effect of aromatase inhibitors (AI) (Fig. 1a). Using endpoint analysis, we previously showed that resistance in this model involves amplification of the aromatase gene (*CYP19A1*) in combination with transcriptional activation of endogenous cholesterol biosynthesis, but not by mutated ESR1^15,28^. Even in this accelerated model, fully resistant cells emerge between 6 and 12 months of oestrogen deprivation^29^, which is incompatible with clonal selection of a pre-resistant cell^30^. In line with this, <1.5% of early-stage BCa show evidence of a pre-existent ESR1-mutant clone^31^ (Supplementary Table 1), suggesting key driver mutations to be acquired at a later stage. However, this model does not fully exclude pre-existence of transcriptomic clones with features of resistance. To investigate this, we generated scRNA-seq high-quality profiles for >1200 MCF7 and >1900 LTED cells (Supplementary Table 2).

Dimensionality reduction (Similarity Weighted Nonnegative Embedding, or SWNE)^32^ showed MCF7 and LTED as completely separated populations, with no single MCF7 clustering with LTED cells (Fig. 1b). Studies in melanoma and TNBC suggest that drug-resistant cells can rapidly emerge^25,26^. This implies that in drug-naive tumours, at least a few cells have a transcriptional profile similar to that of fully resistant cells. However, our data suggest this is not the case in luminal breast cancer cell lines, which is concordant with the long latency taken by resistance to occur in most patients treated with ET. To completely exclude any contribution of a pre-existent genetic clone, we inferred single-cell, copy number alterations (CNAs) from scRNA-seq data (see Methods). Clustering of single MCF7 and LTED cells based on the inferred patterns of CNAs identified two clades, one including all the MCF7 and one all the LTED cells (Fig. 1c). In line with *CYP19A1* significantly contributing to AI resistance in vivo and in vitro^15^, an amplification involving the region was found in LTED cells, but not in MCF7 (Fig. 1c). This was confirmed by shallow whole-genome sequencing (Supplementary Fig. 1a). Clustering of single-cell profiles identified five distinct groups (two for the MCF7 and three for the LTED), mainly driven by differences in cell cycle (Fig. 1d). Even after running the dimensionality reduction step separately on cells assigned to the same cell-cycle phase, MCF7 and LTED cells were unambiguously separable (Supplementary Fig. 1b). Importantly, scRNA-seq confirmed that previously reported pathways, such as cholesterol biosynthesis^27^, are profoundly reprogrammed by ET (Fig. 1d; Supplementary Fig. 1c, d).

Taken together, these data support that AI resistance is not driven by a pre-resistant clone (whether genetic or in a particular transcriptional state), suggesting a multi-step adaptation process in which the necessary hits occur with a different timing during ET. Nevertheless, we could not exclude the presence of a rare, transcriptionally defined clone at a very low frequency. This led us to leverage previously acquired knowledge on cancer cell plasticity to further dissect the phenotypic heterogeneity of cells in the drug-naive condition.

### Phenotypic heterogeneity of luminal breast cancer cells

**Fig. 2:**
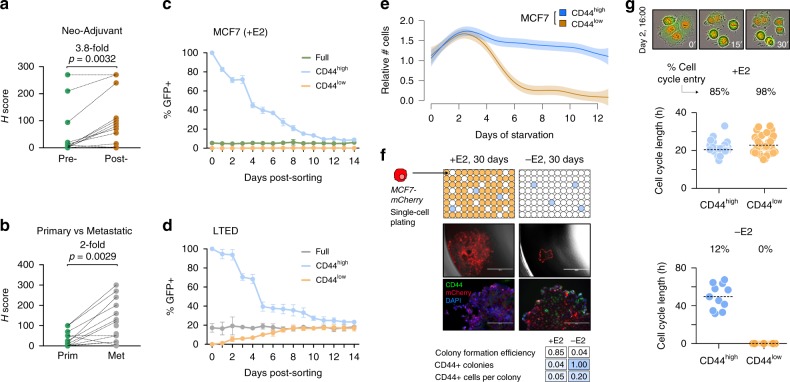
Phenotypic heterogeneity of luminal breast cancer cells. **a** CD44 expression in neo-adjuvant AI-treated patients (pre- and post- treatment; *p*-value from two-tailed paired *t* test). **b** Same as (**a**), but in matched AI-treated primary-metastatic (*p*-value from Wilcoxon signed-rank test). **c** Reconstitution experiments from sorted MCF7-CD44^GFP-high^ or MCF7-CD44^GFP-low^ cells, or the full population. **d** Same as (**c**), but using sorted LTED-CD44^GFP-high^ or LTED-CD44^GFP-low^ cells, or the full population. **e** Survival curves of MCF7-CD44^GFP-high^ and MCF7-CD44^GFP-low^ cells in oestrogen-deprived (-E2) conditions. **f** Single-cell plating experiments in oestrogen-supplemented (+E2) or deprived conditions (−E2) for 30 days. From top to the bottom: (i) schematic representation of the results; (ii) representative pictures of single wells after 30 days (scale bar = 1000 μm); (iii) immunofluorescence staining highlighting CD44 expression (scale bar = 200 μm); (iv) summary statistics. **g** Cell-cycle dynamics of MCF7-CD44^GFP-high^ and MCF7-CD44^GFP-low^ cells inferred from time-lapse imaging. The length of the cell cycle and percentage of cell entering the cell cycle are indicated for both oestrogen-supplemented (+E2; top) and deprived conditions (−E2; bottom)

Previous studies identified CD44 as a marker of plastic cells in various solid tumours^33–35^. It has been suggested that CD44-positive cells possess increased tumorigenic ability and resilience to pharmacological treatments. To investigate the potential role of CD44 as a surface marker to guide the dissection of the phenotypic heterogeneity of luminal breast cancer cells, we identified those genes showing high transcriptional variability across single MCF7 cells (*n* = 778) and intersected them with annotation from the Cell Surface Protein Atlas^36^. CD44 was indeed found among these genes (*n* = 27; Supplementary Fig. 2). Further investigation confirmed variable expression of CD44 also in primary tumours (Supplementary Fig. 3a–c; Fig. 2a, b). Cells expressed significantly higher levels of CD44 after neo-adjuvant AI treatment (Fig. 2a; 3.8-fold, *p*-value = 0.0032; two-tailed paired *t* test) as well as when comparing matched AI-treated primary-metastatic samples (Fig. 2b; Supplementary Fig. 3d; twofold, *p*-value = 0.0029; Wilcoxon signed-rank test), suggesting higher chances of survival to ET for cells expressing CD44 in vivo. We next sought to investigate if CD44^high^ cells can be also found at other active sites in breast cancer patients. Interestingly, we found substantial CD44^high^ cells in pleural effusions from all four patients examined (Supplementary Fig. 3e). In line with this, the fraction of CD44^high^ cells was significantly increased in LTED (upper panels in Supplementary Fig. 3f, g). Extensive functional characterisation of these cells demonstrated that MCF7-CD44^high^ cells were more invasive, more clonogenic and could form first- and second generation of mammosphere at higher efficiency than CD44^low^ cells (Supplementary Fig. 3j–l). In agreement with previous studies^35^, CD44^high^ cells also showed cellular plasticity as they could recapitulate the entire population, while CD44^low^ were capable of generating only CD44^low^ cells (Supplementary Fig. 3f).

To further investigate the plasticity of CD44^high^ cells in vitro at the single-cell level, we generated MCF7 and LTED cell lines with a GFP reporter expressed under the promoter of the *CD44* gene (Supplementary Fig. 4). Reconstitution experiments from sorted cells showed that CD44^GFP-high^ cells could recapitulate all the functional aspects of endogenous CD44^high^ cells, including cellular plasticity (Fig. 2c). Interestingly, both CD44^GFP-high^ and CD44^GFP-low^ showed features of plasticity in fully resistant cells (Fig. 2d; Supplementary Fig. 3g). When MCF7 were challenged with short-term ET, only CD44^GFP-high^ cells appeared to adapt to it, while CD44^GFP-low^ cells were rapidly cleared out between days 4 and 7 (Fig. 2e). Single-cell plating experiments confirmed that only CD44^high^ cells could drive the formation of early colonies under E2 deprivation, but the colonies were significantly smaller compared with E2-supplemented conditions (Fig. 2f). These observations indicate combined cytostatic and cytotoxic effects of ET and that those cells that could adapt to the therapy originate within the CD44^high^ compartment. Extrapolation of cell-cycle dynamics of CD44^GFP-high^ and CD44^GFP-low^ cells from time-lapse imaging data revealed comparable cell-cycle length in E2-supplemented condition (Fig. 2g; +E2). Nevertheless, CD44^GFP-high^ cells had a significantly lower proportion of cells engaged in productive cell-cycle entry, suggesting the existence of a low-proliferative subpopulation within the CD44^high^ compartment even under permissive environments. Under E2 deprivation, the CD44^GFP-low^ completely failed to undergo cell-cycle entry, while 12% of CD44^GFP-high^ managed to do one or more cell cycles, with a much longer latency (Fig. 2g; −E2).

Taken together, these results further support the idea that at least some of the cells in the CD44^GFP-high^ (but not CD44^GFP-low^) compartment have an increased ability to survive the acute phase of ET, and this correlates with their features of plasticity. This led us to hypothesise that non-genetic, transcriptional variability would reflect pre-existent, rare subpopulations in treatment-naive cells with higher chances to survive and give rise to fully resistant cells.

### Transcriptional heterogeneity of plastic cells

**Fig. 3:**
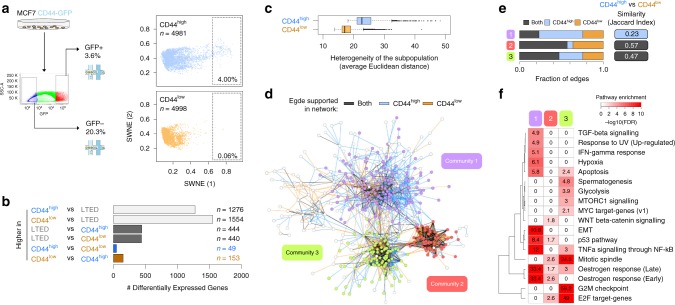
Single-cell transcriptomics reveals heterogeneity of plastic cells. **a** Schematic representation of the strategy to sort MCF7-CD44^GFP-high^ (CD44^high^) and MCF7-CD44^GFP-low^ (CD44^low^) cells (left) along with the results of dimensionality reduction for single-cell transcriptomes (right) (SWNE, *k* = 22); percentage of extreme outliers in the two subpopulations indicated in the bottom right corner. **b** The number of upregulated genes in the indicated comparisons ( *q*-value <= 0.05; AUC >= 0.6). **c** Cell–cell heterogeneity within CD44^high^ and CD44^low^ subpopulations. Box plots show median, interquartile values, range and outliers (individual points). **d** Regulatory networks reconstructed using either CD44^high^ and CD44^low^ profiles were superimposed and the edges colour-coded according to whether each edge was identified only in the CD44^high^ (blue), the CD44^low^ (orange) or both (dark grey) networks. Nodes in the three larger communities were colour-coded accordingly. **e** Fraction of edges identified in the CD44^high^, the CD44^low^ or both networks, for each one of the three communities shown in (**d**). Similarity between CD44^high^ and CD44^low^ networks is also shown. **f** Enrichment analysis using the hallmark gene sets^38^ across the three communities shown in (**d**)

To investigate the transcriptional variability of CD44^high^ cells, we carried out sorting driven, scRNA-seq of CD44-GFP luminal breast cancer cells. About 10,000 single cells in E2-supplemented condition were profiled (CD44^GFP-high^ and CD44^GFP-low^ in equal proportions; Fig. 3a; in the remainder of the text, these two sorted subpopulations will be referred to as CD44^high^ and CD44^low^). Dimensionality reduction (Fig. 3a) highlighted a surprising similarity between the profiles of CD44^high^ and CD44^low^, except for a small percentage (~4%) of CD44^high^ cells significantly departing from the main cluster. In line with this, differential expression analysis of the two subpopulations resulted in tenfold less differentially expressed genes (DEGs) than those observed by comparing them to LTED (Fig. 3b; Supplementary Data 1). Nevertheless, CD44^high^ showed an overall, significantly higher transcriptomic variability (*p*-value < 2.2e-16; Wilcoxon rank-sum test) than CD44^low^ (Fig. 3c).

We next sought to systematically address whether the observed variability was the result of either an increased transcriptional noise specific to CD44^high^ cells (compatible with a bet-hedging mechanism) or instead the reflection of a regulated network (leading to coordinated expression of multiple genes in the same cell). We applied PIDC^37^, an algorithm using partial information decomposition (PID), to identify regulatory relationships between genes, and reconstructed the gene regulatory networks (GRNs) from the scRNA-seq profiles of CD44^high^ and CD44^low^ cells, separately (Supplementary Data 2). The two networks were merged and analysed to identify major communities (Fig. 3d; the three largest communities were consistently identified on the separate CD44^high^ and CD44^low^ networks, with >95% overlap with the corresponding community from the merged network; Supplementary Table 3). The largest of the three identified communities (#1 in Fig. 3d–f) showed the lowest similarity between the CD44^high^ and CD44^low^ GRNs, with the majority of edges supported only by the CD44^high^ GRN (Jaccard Index = 0.23, still higher than expected by chance, expected value = 0.0154,  *q*-value < = 1e-3; Fig. 3e and Methods). Pathway enrichment analyses^38^ for the genes in this community showed highly significant enrichments for oestrogen response, TNFα signalling, epithelial–mesenchymal transition and the p53 pathway (Fig. 3f; *q*-value < 1e-8). These results strongly suggest that the variability specific to the CD44^high^ compartment is the result of the coordinated regulation of genes in a small fraction of cells. With this in mind, we hypothesised a central role for these rare cells in the early phases of acute oestrogen deprivation (termed acute-ET).

### Single-cell transcriptomics identifies pre-adapted cells

**Fig. 4:**
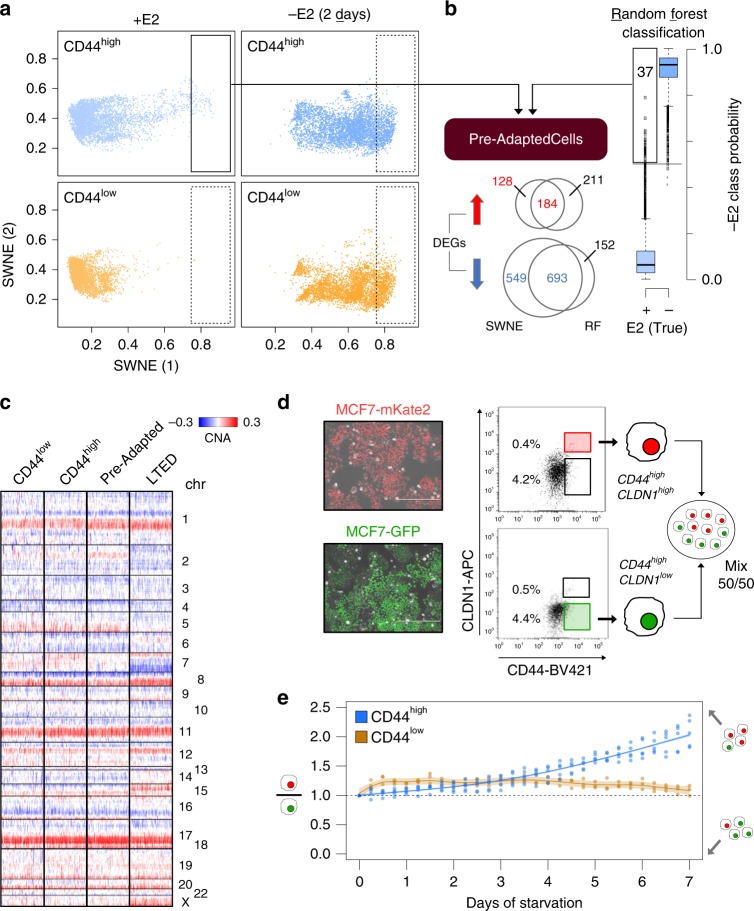
Single-cell transcriptomics identifies pre-adapted cells. **a** Dimensionality reduction of single-cell transcriptional profiles of oestrogen-supplemented (+E2; top) or deprived (−E2; 2 days) cells. Pre-adapted (PA) cells highlighted in boxes. **b** PA cells identification using two different strategies (SWNE and Random Forests). DEGs:  differentially expressed genes. Box plots show median, interquartile values, range and outliers (individual points). **c** Copy number profiles of PA cells (*n* = 81), along with the same number of LTED, CD44^low^ and CD44^high^ (not PA) cells, as estimated from scRNA-seq profiles. **d** Sorted PA cells (CD44^high^ and CLDN1^high^) stably labelled with mKate2 were mixed with other plastic cells (CD44^high^ and CLDN1^low^) stably labelled with GFP (scale bar = 400 μm) and followed up for 7 days upon E2 deprivation (**e**)

To investigate the role of transcriptomic variability of plastic cells during acute-ET, we performed scRNA-seq experiments upon oestrogen deprivation (Supplementary Table 2). Continuous single-cell imaging suggested that cells within the CD44^low^ subpopulation started being differentially affected by acute-ET after 48 h of treatment (Fig. 2e). We thus profiled gene expression data of about 10,000 single cells at 48 h of E2 starvation (Fig. 4a). Applying a stringent threshold on the first SWNE component, we could define a rare, pre-adapted (PA) subpopulation among plastic cells (CD44^high^) expressing a signature of acute-ET even in permissive E2-supplemented condition. The identification of PA cells was confirmed using an orthogonal approach aimed at identifying outliers and based on Random Forests classification (Fig. 4b, Supplementary Data 3 and Methods). We then excluded the PA cells identified using SWNE (Fig. 4a), and trained another Random Forest classifier that was tested on the PA cells (Supplementary Fig. 5a). Overall, 72.8% of PA cells were mis-classified as starved cells, compared with an expected 2.2% (out-of-bag classification error), further corroborating the observation that PA cells are strongly biased towards features of starved cells. Of note, PA cells are genetically indistinguishable from the other CD44^high^ cells, and have not yet acquired any of the genetic re-arrangements of the fully resistant, LTED cells (Fig. 4c). Considering both approaches and either a lenient or a stringent threshold, PA cells are estimated to constitute 0.76–4% of the CD44^high^ cells, which correspond to 0.03–0.14% of the total MCF7 population. Overall, these data suggest that PA cells might represent the first step in the process of adaptation to acute-ET.

We then sought to validate if the PA transcriptional state would confer a survival advantage compared with other plastic cells exposed to acute-ET. First of all, we identified the *Claudin-1* gene (CLDN1) as a suitable surface marker to enrich for PA cells by FACS in combination with CD44 (Supplementary Data 3 and Supplementary Fig. 5b). We then generated MCF7 cells stably labelled with either a nuclear GFP or mKate2 and leveraged this tool to follow two subpopulations over time after mixing them. The same amount of sorted PA cells (CD44^high^ and CLDN1^high^) was mixed with other plastic cells (CD44^high^ and CLDN1^low^; Fig. 4d). CD44^high^ CLDN1^high^ PA cells showed increased survival to acute-ET compared with CD44^high^ CLDN1^low^, with this effect increasing over time. As a control, no difference was observed between CLDN1^high^ and CLDN1^low^ from the CD44^low^ compartment. These data strongly support the hypothesis that PA cells have a distinctive survival advantage under acute-ET (Fig. 4e).

**Fig. 5:**
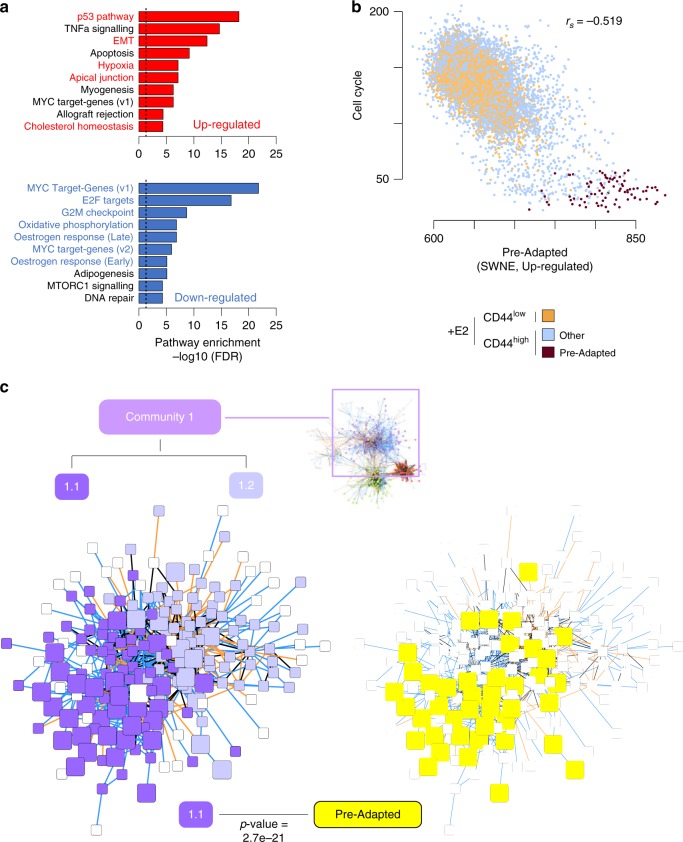
Functional characterisation of the signature of pre-adapted (PA) cells. **a** Hallmark gene sets^38^ enriched in genes either up- or downregulated in pre-adapted (PA) cells. **b** Correlation analysis between expression of cell-cycle marker genes and genes belonging to the PA signature (upregulation) at the single-cell level. r_s_ = Spearman’s rank correlation coefficient. **c** Same representation as in Fig. 3d, but limited to community 1. Two sub-communities were identified (left) with community 1.1 being more strongly enriched for genes in the PA signature (*p*-value from hypergeometric test)

We then further characterised these cells functionally, focusing on the set of differentially expressed genes between the PA cells and the rest of the CD44^high^ cells in +E2 condition (cells identified through the SWNE-based approach; 312 upregulated and 1242 downregulated; Fig. 4a, b; Supplementary Data 3). PA cells displayed features of mixed epithelial and mesenchymal traits, along with upregulation of p53 pathway, cell polarity (apical junction components) and hypoxia (Fig. 5a, upper panel). PA cells also showed reduced ERα activity and downregulation of the cell cycle machinery, while still expressing ESR1 (Fig. 5a, lower panel and Supplementary Fig. 5c). Interestingly, both plastic and non-plastic cells lied on a continuum showing a negative correlation between the expression of the genes of the cell cycle and of those in the signature of PA cells (Fig. 5b; Spearman’s rank correlation coefficient = −0.519; *p*-value < 2.2e-16), with PA cells found at the edge of this spectrum. We finally sought to quantify the overlap between the PA cells signature (upregulated genes) with the CD44^high^-enriched GRN we previously identified (Fig. 3d–f). Indeed, when we further dissected the GRN (community #1) into its two main components, we found extensive overlap between one of these components and the PA signature (Fig. 5c; *p*-value = 2.7e-21; hypergeometric test). This further supports the idea that the genes in this signature are part of a co-regulated network.

Overall, these data support the hypothesis that plastic cells are phenotypically heterogeneous (with no evidence supporting genetic clones), and that among them rare cells in the PA state have a survival advantage during acute-ET.

### PA features persist in acute-ET, but not in full resistance

**Fig. 6:**
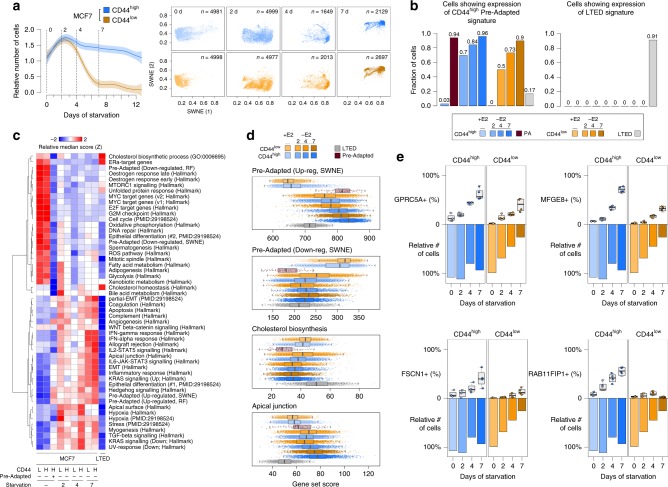
Features of pre-adaptation persist in acute-ET, but not in full resistance. **a** Sampling design along with dimensionality reduction of single-cell transcriptional profiles of E2 supplemented (day 0) or deprived (days 2, 4 and 7). **b** AUCell^39^ quantification of the fraction of single-cells showing transcriptome compatible with either the pre-adapted (left) or the LTED (right) signatures. **c** Selected gene set enrichment across all conditions profiled in this study. **d** Score distributions for the indicated gene sets, across cells. **e** Multi-marker tracing profiles for selected genes (box plots) in CD44^high^ and CD44^low^ cells upon E2 deprivation. Survival (as relative number of residual cells) is also shown (bar plots). Box plots show median, interquartile values, range and outliers (individual points)

While these analyses support a pivotal role for the PA phenotype in conferring a survival advantage during acute-ET, PA cells are still genetically indistinguishable from the rest of the cells. This suggests these cells do not represent the final step of drug resistance. Nevertheless, we aimed at determining whether longer exposure to acute-ET correlates with the persistence of the PA signature, and/or this also coincides with other reprogramming events. In order to capture the different dynamics of survival of CD44^high^ and CD44^low^ (Figs. 2e, 6a), we generated scRNA-seq profiles at 4 and 7 days of E2 deprivation (Supplementary Table 2), a period in which the relative number of CD44^high^ cells does not change while CD44^low^ undergo rapid extinction. Dimensionality reduction of >28 k cells showed increased prevalence of the PA signature with time of starvation (Fig. 6a). Formal quantification using AUCell^39^ confirmed this trend (Fig. 6b, left panel and Fig. 6d). The same analysis using a LTED-specific signature (Methods and Fig. 1) failed to identify any cell expressing it during acute-ET (Fig. 6b, right panel). In line with this, the critical transcriptional pathways driving full resistance (i.e., cholesterol biosynthesis and re-activation of ERα signalling) were completely abrogated in PA and cells exposed to acute-ET (Fig. 6c, d). On the other hand, some of the pathways associated to PA phenotype (partial-EMT, cell polarity, hypoxia) were found to consistently increase during treatment.

Unexpectedly, while imaging showed that after 7 days >75% of the CD44^low^ died and were destined to extinction (Fig. 2f), the profiled CD44^high^ and CD44^low^ cells converged on the same transcriptional changes. We reasoned that since scRNA-seq experiments capture viable cells exclusively, we profiled only those cells that were still alive at day 7. Thus, we hypothesised that the PA-like transcriptional programme is an intermediate bottleneck during acute-ET. In line with this, we discovered that CD44^low^ cells can occasionally upregulate a signature overlapping that of PA cells, but this happens with lower efficiency (Fig. 6b), and it is not sufficient to give them the survival advantage shown by CD44^high^. To validate these observations at the protein level, we performed a multi-marker tracing profile, exploiting some marker genes (namely, GPRC5A, MFGE8, FSCN1 and RAB11FIP1) showing a trend of upregulation with starvation time. This trend was confirmed at the protein level, with values consistently higher in CD44^high^ compared with CD44^low^ cells (Fig. 6e; Supplementary Figs. 6, 7). Nevertheless, this did not prevent cells in the CD44^low^ compartment to die at an almost linear rate (Fig. 6e).

Taken together, these observations confirmed that the PA transcriptome is strongly selected by acute-ET. Nevertheless, the observed rapid expansion (Fig. 6b) seems incompatible with the strict selection of a pre-existing population^30^. The observation that also CD44^low^ cells can adopt a similar transcriptional profile in response to ET (despite being unable to survive) suggests that the PA programme is required, but not sufficient to explain the survival of plastic cells to acute-ET (see Discussion).

### The PA signature is enriched in clusters of CTCs

**Fig. 7:**
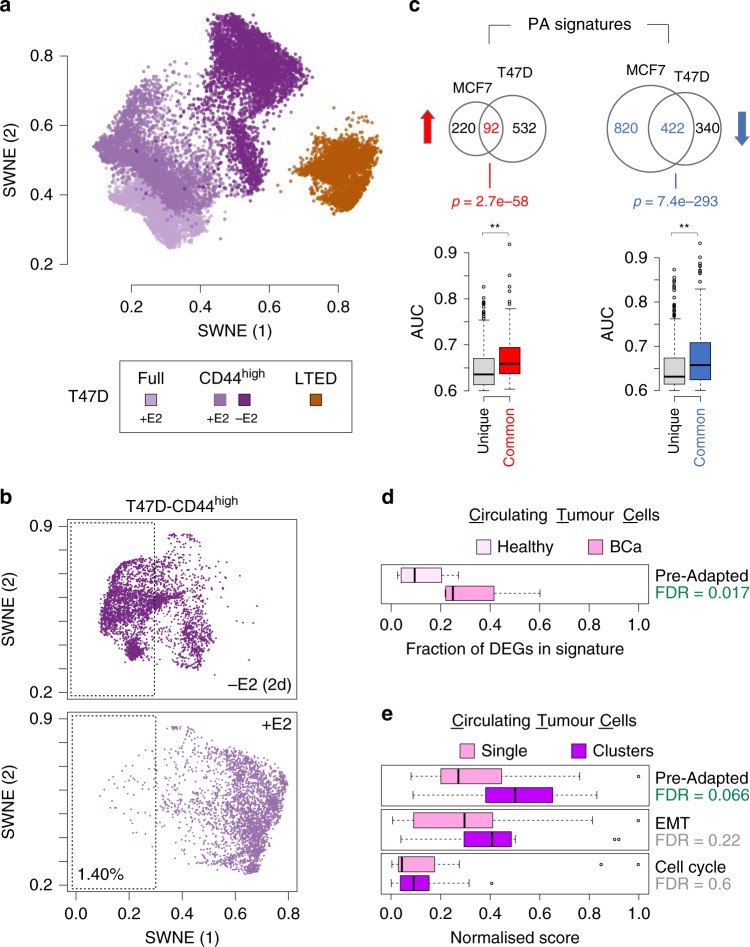
Evidence for pre-adaptation in T47D and circulating tumour cells. **a** Bi-dimensional representation of 15,805 transcriptomes from single T47D and LTED cells (Supplementary Table 2) (SWNE; *k* = 20). **b** Dimensionality reduction of single-cell transcriptional profiles of oestrogen-supplemented (+E2; top) or deprived (−E2; 2 days) T47D-CD44^high^ cells. Pre-adapted (PA) cells highlighted in boxes. **c** (Top) Venn diagrams showing the overlap between the MCF7-PA and T47D-PA signatures (upregulated genes in red; downregulated in blue). *P*-values of the overlaps calculated via hypergeometric test. (Bottom) Box plots indicating the effect size (AUC) of those genes unique to either MCF7- or T47D-PA or common to both. **p <= 1e-5 (Wilcoxon rank-sum test). **d** Expression of the PA signature in circulating tumour cells (CTCs) compared with blood specimens from healthy donors. **e** Expression of the PA, the epithelial-to-mesenchymal transition (EMT) and a cell-cycle signatures in clusters of CTCs compared with single CTCs. False discovery rates estimated by permutations. Box plots show median, interquartile values, range and outliers (individual points)

T47D cells are another widely used model of hormone-dependent breast ductal carcinoma that differ from MCF7 by their TP53 status (mutated in T47D). We first confirmed that CD44 is a bona fide marker of plasticity also in these cells (Supplementary Fig. 3h, i). We then derived treatment-naive T47D cells stably expressing a GFP reporter under the promoter of the CD44 gene. With this tool, we generated high-quality scRNA-seq profiles from sorted T47D-CD44^high^ cells, either in the presence or absence of E2. We also profiled unsorted population of T47D and LTED cells (capturing ~3000–4000 cells each; Supplementary Table 2). In line with what observed with MCF7 (Fig. 1b), dimensionality reduction (SWNE) indicated that no single treatment-naive cell clustered with LTED cells (Fig. 7a; Supplementary Fig. 8a; Supplementary Data 4). We then looked specifically to CD44^high^ cells and, similarly to MCF7 (Fig. 4a), we were able to identify a small fraction of treatment-naive cells overlapping with the E2-deprived cells (Fig. 7b). Up- and downregulated genes in T47D-PA cells showed extensive, highly significant overlap with those singled out in MCF7-PA (Fig. 7c; ~15% and ~55% of the up- and downregulated, respectively; *p* < = 2.2e-16, hypergeometric test), with overlapping genes showing significantly higher effect size compared with the rest (Fig. 7c; *p* < = 1e-5; Wilcoxon rank-sum test). Importantly, CNAs estimated from scRNA-seq data sets support the idea that also T47D-PA cells are transcriptional clones. Although these cells do not show genetic lesions of LTED cells (Supplementary Fig. 8b), the estimated profiles are qualitatively more heterogeneous than those of the other plastic cells (Supplementary Fig. 8c). These results further strengthen a role for the identified PA signature in the survival to AI.

We then looked for evidence of expression and co-regulation of genes upregulated in PA cells, in 825 primary luminal breast tumours^40^. Tumours classified as luminal A showed significantly higher expression of the signature compared with luminal B (*p*-value < 2.2e-16; Wilcoxon rank-sum test) and TNBC/HER2 + lesions (*p*-value = 1.9e-8) (Supplementary Fig. 8d). Of note, luminal A exhibits the longest latencies in relapse development amongst all BCa^41–43^. Considering > 600 luminal A samples, we then checked the distribution of pairwise correlations between the expression pattern of the genes in the signature, as a proxy for co-regulation. Compared with a size-matched set of randomly picked genes, those in the PA signature showed significantly higher coefficients (Supplementary Fig. 8e; *p*-value < 2.2e-16; Wilcoxon rank-sum test), with hundreds of pairs with values over 0.5 (Spearman’s rank correlation coefficient). These results further corroborate our previous observations that these genes tend to be controlled by the same GRNs, and showed a trend of higher expression in luminal tumours with longer latency of recurrence (A vs B; Supplementary Fig. 8d).

Given that some of the key pathways active in PA cells hinted to mixed epithelial and mesenchymal features, as well as cell polarity and migration, we asked if the PA phenotype could play a role in metastatic progression. Previous data strongly suggest that epithelial-like clusters of circulating tumour cells (CTCs) are responsible for 85–92% of metastatic dissemination^44^, with individual CTC showing more mesenchymal features playing a more limited role^45^. Interestingly, the PA signature was found significantly enriched in CTCs^45^ (Fig. 7d; *q*-value = 0.017, permutation test) and at even higher levels in clusters of CTCs^44^ (Fig. 7e; *q*-value = 0.066, permutation test). These results provide a further link between drug-induced adaptation and metastatic invasion^27,46^.

## Discussion

In this study, we leveraged two in vitro models to investigate the contribution of genetic and transcriptional heterogeneity to the development of resistance to ET in luminal breast cancer. As opposed to previous observations in melanoma, TNBC, lung and colorectal cancers, in which targeted therapy lead to the rapid emergence of fully resistant cells^10–12,18,26^, we could not find any genetic or phenotypic clone showing features of resistance in treatment-naive cells (Figs. 1, 7). The same observation held true even after thoroughly dissecting the heterogeneity of the cells showing features of plasticity (Figs. 2, 3 and 7). On the other hand, we could identify and characterise a small subpopulation (~0.1% of the treatment-naive cells) showing a PA phenotype (Figs. 4, 7). These cells showed a twofold increased survival to acute-ET compared with other plastic cells (while non-plastic cells undergo complete extinction under selective pressure; Fig. 4e), along with mixed epithelial and mesenchymal features, and quiescence. Interestingly, while any cell (also those with no feature of plasticity) can adopt a transcriptional programme overlapping that of the PA cells, only plastic cells can withstand acute-ET (Fig. 6a, e), with PA cells showing a more pronounced survival advantage (Fig. 4e). Finally, we found an enrichment of the PA signature in clusters of CTCs, linking a quiescent subpopulation from the primary tumour to both features of survival to therapy and of CTCs. Interestingly, it has been reported that early-stage metastatic cells possess partial features of survival, dormancy and EMT, which all overlap with our PA signature^47^. A signature of partial-EMT has also been recently shown to be expressed in the cells at the leading edge of primary head and neck cancers^48^. It is tempting to speculate that PA cells might not only display a survival advantage during the early phases of the therapy but might also be the pioneers of micro-metastatic spread.

Surprisingly, we found that also cells with no features of plasticity were able to adopt the PA signature, even though with a much lower efficiency, which cannot prevent the extinction of the compartment after two weeks of E2 deprivation (Fig. 6). On top of this, 70% of the plastic cells adopted a PA signature within 48 h of acute-ET (Fig. 6b). This fast transition to a diverse transcriptional state is hardly explained by conventional Darwinian selection of a pre-resistant (or persister) cell^30^. For reasons that remain to be investigated, plastic cells have a much higher probability than non-plastic ones to transition into a PA state, and this probability is dramatically increased by E2 deprivation. We reason that upon stress, plastic PA cells are better positioned than cells requiring transcriptional reprogramming, hence the observed difference in survival within the plastic compartment (Fig. 6e). We estimated PA cells to constitute ~0.1% of the treatment-naive cells. In order to obtain ~100 PA cells would have required profiling at least 70,000 MCF7 cells by scRNA-seq. Even in the best-case scenario, this single experiment requires capturing more cells than those profiled across this entire study (Supplementary Table 2). This suggests that functional approaches leading to dissection of the phenotypic heterogeneity, and thus to enrichment strategies (Fig. 2) are required for the feasibility of this kind of studies.

**Fig. 8:**
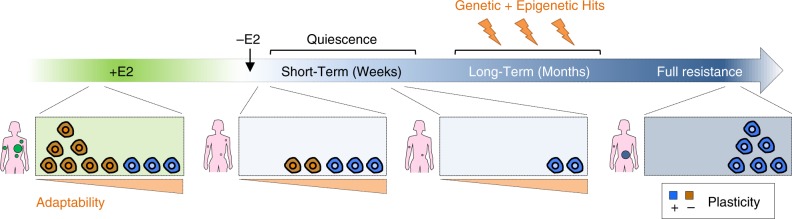
Proposed multi-step model of resistance to endocrine therapies. (Left to right). A hierarchy of cells with or without features of plasticity (in blue and brown) co-exist in the primary tumour and in the micro-metastases (large and small green circles, respectively). These cells are already positioned on a gradient of probability to survive to future exposure to endocrine therapies (light green box). Upon surgery and start of adjuvant treatment (−E2), only a handful of plastic cells is able to survive (light blue boxes). These cells enrich for the transcriptional signature of pre-adaptation identified in this work, and in turn are those able to accumulate those genetic hits and further transcriptional re-wiring observed in the fully resistant cells, which eventually lead to metastasis (large blue circle)

The data presented here suggest PA cells as an obligated step towards the acquisition of resistance while still requiring substantial reprogramming to recapitulate features of fully resistant cells (Fig. 8). We propose that the delayed relapse common to ET-treated patients might be mediated by similar processes, in which PA-like cells are selected for and stalled by ET for up to >10 years. This model would reconcile why ET are sometime effective for downstaging neo-adjuvant patients, but fail to clear micro-metastatic disease. Nevertheless, single-cell lineage-tracing approaches coupling unambiguous identification of clones to transcriptome mapping are needed to get a definitive proof that it is the progeny of PA cells that will eventually acquire full resistance. Besides, how this bottleneck affects the progression of the tumour requires further investigation. Future studies on the necessary steps and their timing of occurrence during treatment must be carried out in order to expose potential vulnerabilities of these quiescent cells.

## Methods

### Cell lines

MCF7 and long-term oestrogen-deprived cells (LTED) were kindly provided by Philippa Darbre and T47D and LTED were kindly provided by Matthew Ellis^27^. MCF7 and T47D cells were maintained in the Dulbecco’s modified Eagle’s medium (DMEM) containing 10% foetal calf serum (FCS) Long-term oestrogen-deprived cells (LTED) were derived from MCF7 or T47D after 1 year oestrogen deprivation and were maintained in phenol-red free DMEM containing 10% charcoal stripped foetal calf serum (SFCS)^27^. Both media were supplemented with 2 mM L-glutamine, 100 units/mL penicillin and streptomycin. 10^−8^ M oestradiol (E2758 Sigma) was added routinely to MCF7. Primary-metastatic breast cancer cells were derived from pleural effusions of patients with metastatic breast cancers. The pleural effusion (PE) cells were maintained in the DMEM containing 10% foetal calf serum (FCS) and 2 mM L-glutamine, 100 units/mL penicillin and streptomycin. Written informed consent for the procedure was obtained from all patients. The study was reviewed and approved by Imperial College Healthcare NHS Trust Tissue Bank (R14059). Cells were tested for mycoplasma contamination before the experiments and showed negative results.

### Plasmids

pLVX-IRES-mCherry-puro lentiviral vector (Cambridge Bioscience, Cambridge, UK) was used to infect MCF7 and LTED cells. MCF7- and T47D-CD44 reporter GFP cells were established with CD44CR1-IRES-GFP-puro lentiviral vector (Tebu-Bioscience). Stable and polyclonal cell populations were established after puromycin selection (0.5 μg/ml). NucLight Green lentivirus (IncuCyte, 4626) and NucLight Red Lentivirus (IncuCyte, 4627) were used to infect MCF7. Stable and polyclonal cell populations were established after Zeocin selection (300 μg/ml). H2B-mCherry-puro lentiviral vector was used to infect stable CD44 reporter GFP cells. Stable and polyclonal cell populations were established after sorting.

### Antibodies

Anti-ERα antibody (Vector Laboratories, VP-E613) 1:100 for immunofluorescence (IF) and anti-ERα (Santa Cruz, HC-20) 1:1000 for western blot (WB), anti-CD44 antibody (Santa Cruz, sc-7297) 1:200 for IF and 1:100 for immunohistochemistry (IHC), anti-pan Cytochemistry antibody (Abcam, ab17154) 1:200 for IF, anti-FGFR4 antibody (Abcam, ab44971) 1:100 for IF, anti-FSCN1 (Sigma, HPA005723) 1:100 for IF, anti-MFGE8 (Sigma, HPA002807) 1:100 for IF, anti-RAB11FIP1 (Sigma, HPA023904) 1:100 for IF, anti-GPRC5A (Sigma, HPA007928) 1:100 for IF and anti-caspase3 (Merk, MAB10753) 1:100 for IF.

### FACS analysis

Cells were cultured to 70–80% confluence and detached from the cell culture flasks using EDTA. Cell pellets were obtained and washed with cold phosphate-buffered saline (PBS) containing 1% FCS and 5 mM EDTA. All further steps were performed on ice and all centrifugation steps at 4 °C. Fluorochrome-conjugated monoclonal antibodies against human CD44 (FITC, BD Pharmingen; BV421, BD Pharmingen), Claudin-1 (APC, R&D systems), and their isotype controls were added to the cell suspension at concentrations recommended by the manufacturer (BD Biosciences) and incubated at 4 °C in the dark for 30 min. The labelled cells and CD44 reporter GFP cells were washed in PBS and then were analysed on a FACS Aria (BD Biosciences). Gating was set to relevant isotype control (IgG-FITC)-labelled cells or unstained cells for each cell line. Propidium iodide (Bio-Rad, 1351101) and DRAQ7 (BioLegend, 424001) were used for the dead cell removal.

### Soft agar colony-forming assay

Anchorage independent cell growth was carried out in six-well tissue culture plates. A 1-mL layer of 0.6% agar (DIFCO Laboratories) in appropriate cell culture medium was solidified at the bottom of each well. Cells to be assayed were suspended in 1 mL of 0.3% agar in medium. In all, 1 × 10^4^ cells were seeded in each dish. After 4 weeks of incubation at 37 °C in 5% CO_2_, colonies were visualised by staining with 0.02% crystal violet.

### Mammosphere culture

Cells were plated as single cells at a density of 5 × 10^2^ viable cells/well in ultralow attachment six-well plates (Corning, CLS3814). Cells were grown in a serum-free DMEM or phenol-red free DMEM, supplemented with B27 (Invitrogen, 17504-044), 20 ng/mL EGF (Sigma, E9644) and 20 ng/mL bFGF (R&D systems, 233-FB-025). Mammospheres were grown for 10–14 days, and phase contrast images were obtained using the ImageXpress Micro microscope (Molecular Devices). For the second-generation experiment, first-generation mammospheres were collected from multiple wells and spun at 500 × g per 5 min. The pellet was resuspended in 50 μl of Trypsin, and the sample was passed 25 times through a sterile needle to get single-cell suspension. The same density of cells as in first-generation culture was seeded, and cells were allowed to grow for 14 days.

### Immunofluorescence

Briefly, 10^4^ cells were seeded on chamber slides (Lab-Tek). On the final day, cells were washed twice with PBS at room temperature, and 4% PFA/PBS was added for 15 min. Cells were washed twice with PBS, and NH_4_Cl was added as a quencher. In all, 0.2% Triton/PBS was added for 5 min. In all, 10% BSA/PBS was used as a blocking reagent. Five percent of BSA/PBS was used to dilute primary antibodies and Alexa-fluor 488, 568, 594, 647 labelled anti-rabbit or anti-mouse secondary antibodies (ThermoFisher). Nuclei were counterstained with DAPI, and were mounted in ProLong Antifade Mountant (ThermoFisher, P36941). Pictures were acquired using the EVOS microscope system (Advanced Microscopy Group, Bothell, WA, USA) or a Zeiss Axiovert 200 M inverted microscope.

### Tissue microarray (TMA)

Twenty primary breast carcinomas with a paired metastasis were acquired from the pathology archives of Charing Cross Hospital, London, UK. A tissue microarray was constructed using a manual microarrayer and 0.6 mm punches. The tissue microarray was immunohistochemically profiled for CD44 (Santa Cruz sc-7297). Antigen retrieval was performed using 0.01 M citrate buffer, pH 6.0 followed by blocking in 0.3% hydrogen peroxide in PBS, then in normal goat serum (20 μl per ml) for 30 min. The primary antibody was incubated overnight at 4 **°**C at 1:100, and then detected using anti-mouse secondary antibody (Vector Laboratories), Vectastain Elite peroxidase ABC kit and ImmPACT DAB kit (Vector Laboratories). Subsequently, 4 μm TMA sections were immunostained using the optimised staining protocol, including negative controls (omission of the primary antibody). Staining was scored based on the H-score by three independent investigators (including one consultant pathologist) blinded to the clinicopathological characteristics of patients. H = (3 × % of strongly stained cells) + (2 × % of moderately stained cells) + (1 × % of weakly stained cells) + (0 × % of cells without staining). Negative controls were performed by omission of the primary antibody. The study was reviewed and approved by Imperial College Healthcare NHS Trust Tissue Bank (R14111).

### Neo-adjuvant treated patient selection

All clinical data from patients operated at the European Institute of Oncology (IEO) were prospectively entered in an Institutional database. For this study, we retrieved data from patients with a neo-adjuvant AI treatment from 1999 to 2014. We selected patients having had pre-surgical biopsy and surgery in our Institute, in order to have their sample analysed in the same laboratory. We randomly selected 20 patients with luminal tumours, treated by neo-adjuvant hormone only (aromatase inhibitors), 10 responders and 10 non-responders. The tissue bank was regulated by the IEO, and written informed consent for collecting samples was obtained from all patients.

### Reconstitution assay

We first sorted cells for endogenous CD44 using FACS Aria III (BD Biosciences), we then seeded 10^5^ cells of CD44^high^ and CD44^low^ on six-well plates with E2 supplement and incubated them for 7 days. After 7 days, cells were trypsinised and stained with anti-CD44 antibody (FITC, BD) for FACS. In order to follow plasticity in real time, we sorted MCF7- and LTED-CD44-GFP cells for GFP expression using FACS Aria III (BD Biosciences). Overall, 10^5^ cells of CD44^GFP-high^ and CD44^GFP-low^ were seeded on six-well plates with E2 supplement or deprivation. Five pictures per condition were taken using an EVOS microscope system (Advanced Microscopy Group, Bothell, WA, USA) for 14 days. Fifty different fields were counted. The percentage of GFP-positive cells was calculated by the number of GFP-positive cells/number of total cells × 100.

### Monoclonal assay

Using FACS Aria III (BD Biosciences), single cell of mCherry-MCF7 was seeded on 96-well plates with E2 supplement or deprivation. Single cell was confirmed using an EVOS microscope system (Advanced Microscopy Group, Bothell, WA, USA). The cells were incubated for 30 days, and colonies were counted on the EVOS microscope system.

### Live cell imaging and data analysis

After sorting with GFP by FACS Aria III (BD Biosciences), 10^5^ cells of H2B mCherry-MCF7-CD44^rep^ GFP^high^ and GFP^low^ were seeded on six-well plates with E2 supplement or deprivation. Time-lapse live cell imaging was performed on IncuCyte ZOOM (Essen BioScience) equipped with temperature, humidity and CO_2_ control. Images were acquired every 6 h with 10× plan fluorescence objectives for a proliferation assay and every 15 min with 20x up to 10 days for a cell-cycle analysis. Excitation (Ex) and emission (Em) filters sets (Chroma Technology Corporation) were as follows: CFP, 427-10 nm (Ex), 483-32 nm (Em); YFP, 504-12 nm (Ex), 542-27 nm (Em); mCherry, 589-15 nm (Ex) and 632-22 nm (Em). Micromanager 1.3 was used for acquisition of time-lapse images. All data analysis was done with scripts written in Matlab (Mathworks) or using Cell Profiler (Broad Institute) and ImageJ (National Institutes of Health). Symmetric/asymmetric/conversion analyses were performed on a total of 200 cells. Each cell was monitored for the first three cell divisions (one cell to two cells, two cells to four cells, four cells to eight cells). Symmetric division was scored if the daughter cell matched the mother. Asymmetric was scored if the daughter cell did not match the mother. Conversion was scored if cell changed CD44 status without cell division (at least 4 h pre- or post division). Cell-cycle speed was established by calculating the time intervening between two consecutive metaphase plates.

### Statistical analyses

Unless specified otherwise, all the analyses and plots were performed in the statistical computing environment R v3 (www.r-project.org).

### Single cell preparation

Single cells were prepared from a full population of MCF7 and MCF7-LTED, or T47D and T47D-LTED. At different time points of E2 deprivation, single cells were prepared from sorted MCF7 or T47D-CD44-GFP reporter cells by the level of GFP expression. After centrifugation, single cells were washed with PBS and were resuspended with a buffer (Ca^++^/Mg^++^ free PBS + 0.04% BSA) at 1000 cells/µl.

### Single-cell RNA sequencing

Viability was confirmed to be >90% in all samples using acridine orange/propidium iodide dye with LUNA-FL Dual Fluorescence Cell Counter (Logos Biosystems, L20001). Single-cell suspensions were loaded on a Chromium Single Cell 3′ Chip (10X Genomics), and were run in the Chromium Controller to generate single-cell gel bead-in-emulsions using the 10x genomics 3′ Chromium v2.0 platform as per the manufacturer’s instructions. Single-cell RNA-seq libraries were prepared according to the manufacturer’s protocol, and the library quality was confirmed with a Bioanalyzer High-Sensitivity DNA Kit (Agilent, 5067-4627) and a Qubit dsDNA HS Assay Kit (ThermoFisher, Q32851). Samples were pooled up to four, and sequenced on an Illumina HiSeq 4000 according to the standard 10X Genomics protocol.

### Single-cell RNA-seq raw data analysis

cellRanger (v2.1.1) was run on the raw data using GRCh38 annotation (v1.2.0). Output from cellRanger was loaded into R using the function *load_cellranger_matrix_h5* from package *cellranger* (v1.1.0; genome = “GRCh38”). Data sets were merged according to gene names. All cells sampled were retained, except for flow-sorted CD44^high^ and CD44^low^ either in +E2 media or starved for 2 days, for which the top 5000 cells in terms of UMIs per cell were considered. In order to robustly detect transcriptional states, a recent paper suggested to consider a coverage of at least 1500 detected genes per cell^49^. A filter on cells showing at least 1500 detected genes per cell, and at least 5000 UMIs per cell was then applied. After that, reads mapping on mitochondrial genes were excluded. Before normalisation, a series of filtering steps were performed. To do that, data were imported in *Seurat* (v2.3.4)^50^ and scaled (*NormalizeData* function using normalisation.method = “LogNormalize”, scale.factor = 10,000, followed by the *ScaleData* function). A filtering step was then performed based on the cumulative level of expression (the sum of the Seurat-scaled values) of three housekeeping genes (GAPDH, RPL26 and RPL36)^51^. Manual inspection of these values versus the number of UMIs per cell (or the number of genes with non-zero expression per cell) revealed no correlation between the two. Nevertheless, a number of cells showed very low expression for these genes. Cells showing housekeeping gene expression in the bottom 1% were then excluded from further analyses. At last, genes expressed in less than 20 cells were excluded. Across cells normalisation was performed using the R package *Scran* (v1.6.9)^52^. Raw counts were imported into a SCE object using the *newSCESet* function; size factors were calculated using *computeSumFactors* (sizes = seq(20, 250, 10)), on data pre-clustered through *quickCluster*.

### Estimation of copy number alterations from scRNA-seq data

CNAs were estimated directly from the scRNA-seq data, using an approach similar to the one used by Patel et al.^53^. Only genes expressed in >= 25 cells were considered.

A reference gene expression profile was generated based on published scRNA-seq profiles of hormone-responsive luminal cells (termed L2)^54^, using only the data sets obtained using a droplet-based approach. After normalising each single-cell profile based on the total number of detected transcripts to a fixed constant (10,000), a pseudo-bulk profile for the L2-cells was derived using the mean expression value of each gene across all cells.

Before running the actual CNAs quantification, all the raw scRNA-seq data sets generated in this study (after filtering, pre-normalisation) and the pseudo-bulk profile generated as described above were linearly normalised to a constant (10,000) and log-scaled (pseudo-count set to 1).

First of all, chromosomal coordinates of all genes were retrieved using the *biomaRt* R package (v2.34.2; host set to “jul2015.archive.ensembl.org”)^55^. This way, genes were sorted by chromosomal coordinates. A genome-wide scan was then conducted using a sliding window of 100 genes, with a step of 10. Using the *rollapply* function from the *zoo* package in R (v1.8-3), mean value of expression in each bin was calculated for each single cell, as well as for the reference profile. The resulting genome-wide profile from each single cell was then linearly regressed against the reference estimate (using the function *lm*). The residuals were then considered as a proxy for CNAs and plotted in the form of heat maps. Single-cell CNAs profiles were hierarchically clustered (hclust, method = “ward.D2”) and shown as a circular dendrogram using *circlize_dendrogram* from R package *dendextend* (v1.8.0). In case of full populations, CNAs were estimated on all the cells. In case of the identified pre-adapted cells, the same number of cells was randomly sampled from the other groups of cells.

### Estimation of copy number alterations from ChIP-input-DNA

Reads were aligned to the hg19 human reference genome using bowtie2 (v2.3.4.3)^56^. Aligned reads were converted to BAM files, sorted and indexed using Samtools (v1.9)^57^. Duplicated reads were marked and removed using Picard *MarkDuplicates* (v2.1.1; REMOVE_DUPLICATES = true). Only uniquely mapped reads were retained for further analyses. Copy numbers were inferred using CNVkit tools (v.0.9.4.dev0)^58^, as described here: https://cnvkit.readthedocs.io/en/stable/pipeline.html. CNVkit was run with the default parameters of the *batch* command after creating a flat reference genome as suggested in the manual using the command *reference*.

### Dimensionality reduction and clustering

Normalised data were then imported in *Seurat* and scaled. Variable genes were identified using the *FindVariableGenes* function (mean.function = ExpMean, dispersion.function = LogVMR, x.low.cutoff = 0.01, x.high.cutoff = 6, y.cutoff = 0.01, num.bin = 100). Principal component analysis (PCA) was run using variable genes as input, and the top 50 components were kept. Clusters were then identified using *FindClusters* (resolution = 0.6). Considering only those variable genes identified, as described above (Similarity Weighted Nonnegative Embedding (SWNE)^32^, was applied to further reduce the dimensionality of the data. The *k* parameter was estimated using *FindNumFactors* on a subsample of 1000 cells (loss = “mse”, 2–50 as range of values, with a step of 2). The choice of *k* is determined by randomly set 20% of the gene expression matrix as missing, followed by finding the factorisation that best imputes the missing values, minimising the mean squared error. Using this parameter, nonnegative matrix factorisation was then run through *RunNMF* (alpha = 0, init = “ica”, loss = “mse”), followed by *EmbedSWNE* (alpha.exp = 1.25, snn.exp = 1.0, n_pull = n_4, dist.use = “IC”). For this step, the shared nearest neighbour (SNN) matrix calculated by the *FindClusters* function of *Seurat* was used.

### Differential expression analysis

The two-sample Likelihood Ratio Test implemented in the *LRT* function of the *MAST* R package (v1.4.1)^59^ was used to identify marker genes for a given sample or cluster. Briefly, each cell was either flagged as either belonging to the sample (or the cluster) or not. Those genes identified as upregulated in the cluster at  *q*-value <= 0.05 (Benjamini–Hochberg correction)^60^ and showing an area under the curve (AUC) >= 0.6 were classified as markers for the sample or the cluster. The AUC is an estimate on how accurately a certain gene predicts a cell as part of a certain sample or cluster. AUCs were calculated using the *ROCR* R package^61^.

### Functional enrichment analyses

For functional enrichment analyses, a selected number of gene sets was employed. The 50 Hallmark gene sets from the Molecular Signature Database (MSigDB)^38^ were downloaded from the MSigDB website October 19, 2017. Gene sets from Puram et al.^48^ (Table S7), along with a manually curated list of ERα-core target genes (BYSL, GREB1, HEY2, MPHOSPH10, MYB, NIP7, RARA, SLC9A3R1, TFF1, XBP1) were also considered. For a given subset of cells, each gene set was scored separately as the sum of the normalised expression values of all the genes in the set. The resulting distributions were then used for statistical testing and visualisation.

### Single-cell gene regulatory network inference

Networks were inferred separately for CD44^high^ and CD44^low^ cells, with nodes representing genes and edges representing statistical dependencies between gene pairs. For each data set, genes expressed in fewer than 20% of cells were excluded; then all possible network edges were ranked using the PIDC network inference algorithm^37^ implemented in NetworkInference.jl (http://github.com/Tchanders/NetworkInference.jl), with expression data for each gene discretised independently into 6 bins of equal width; finally, a network was defined keeping the 2000 highest ranking edges. The two networks were then superimposed to form an overlapping network with edges belonging (i) only to the CD44^high^ network, (ii) only to the CD44^low^ network, or (iii) to both networks. Communities were detected in the overlapping network (and recursively in each community) using the label propagation method implemented in LightGraphs.jl (http://github.com/JuliaGraphs/LightGraphs.jl). Communities were required to include at least ten nodes. Similarity of the CD44^high^ and CD44^low^ networks within each community was calculated using the Jaccard index: the number of edges in the community that belong to both the CD44^high^ and CD44^low^ networks divided by the total number of edges in the community; an edge was deemed to belong to a community if it connected two nodes in the community. In order to estimate the probability of getting an equal or higher similarity value by chance, we first generated 1000 random configuration models with the same degree distribution of a given community, separately for the CD44^high^ and CD44^low^-derived networks. The Jaccard similarities of each randomly generated pair was then used to build a null distribution from which empirically estimate a *p*-value. The mean of this distribution was considered as the expected value.

### Identification of pre-adapted cells

Two different strategies were employed to identify the pre-adapted cells. The first one takes advantage of SWNE; a threshold was applied on the first component and the cells showing extreme values (>=0.75) were labelled as pre-adapted. The second strategy leverages random forests classifiers^62^. First of all, the data sets of CD44^high^ cells in +E2 media and starved conditions (2 days) were split into training and testing sets, using 10% and 90% of the cells, respectively. The training set was then used to call the DEGs between the two conditions (+E2 vs starved), using the procedure described in the *Differential expression analysis* paragraph above. These DEGs were used as input features to train a random forest classifier, using the *randomForest* R package (v4.6-14; default parameters). This model was then used to test the remaining data. Those cells in the testing set labelled as +E2 that were showing a probability >50% of being classified as starved were considered pre-adapted.

AUCell^39^ (R package v1.0.0) was the used to quantify the activity of the pre-adapted signatures (and of other signatures, whenever indicated in the text) in single cells. First of all, normalised data were processed using the *AUCell_buildRankings* function. The resulting rankings, along with the signatures of interest, were then subject to function *AUCell_calcAUC* (aucMaxRank set to 5% of the number of input genes). Following inspection of the resulting distributions, thresholds were then manually set to 0.37, 0.18 and 0.32 for the signatures of pre-adapted cells either based on SWNE or random forests, or for the LTED signature (defined as those genes upregulated in LTED *vs* MCF7, as described in the Differential expression analysis section above).

### Re-analysis of published primary samples

Bulk RNA-seq data sets for 1222 breast cancer samples were downloaded from the GDC (Genomic Data Commons)^40^ data portal (http://portal.gdc.cancer.gov/) using *gdc-client*, according to metadata obtained on July 25, 2018. Gene features were normalised to sequencing depth. Given that only a fraction of the samples was pre-classified using PAM50^63^, *k*-nearest neighbours (*k*-NN) classification was employed to impute the rest of the samples. This was performed via the *knn* function in the R package class (v7.3-14), using the pre-classified samples as the training data. Unclassified samples were ascribed to a particular subtype only when showing >60% probability of being assigned to that class. Spearman’s correlations between expression profiles of pairs of genes were calculated on the depth-normalised values. Prior to calculating signature scores, these numbers were further log2-transformed (pseudo-count set to 1) and scaled to *z*-score gene-wise.

### Re-analysis of published profiles of CTCs

Normalised data for circulating tumour cells (CTCs) collected at five time points from a single patient along with identically processed blood specimens from 10 healthy donors^45^ were downloaded from GEO (GSE41245). For each capture, the log2-fold-change between EPCAM+ cells and the matched IgG+ cells (control) was calculated. DEGs were defined as those genes showing a linear fold change between EPCAM+ cells and control >= 1.5. The fraction of DEGs overlapping the genes in the pre-adapted signature was then calculated for each pair. To test if the observed difference between the fraction of DEGs in CTCs and in healthy specimens was random, a P-value was calculated using the Wilcoxon rank-sum test. The corresponding false discovery rate (FDR) was estimated by 1000 permutations.

Raw data for individual CTC-clusters (median of three cells per cluster) and numerically matched pools of single CTCs from the same specimen^44^ were downloaded from GEO (GSE51827). Each profile was normalised by depth, then a profile-specific score was derived for the signature of the pre-adapted cells by summing the normalised expression values of all genes in the signature. These numbers were then divided by the maximum across all profiles. To test if the observed difference between the values obtained for the clusters against the matched pools of CTCs, a P-value was calculated using the Wilcoxon rank-sum test. The corresponding false discovery rate (FDR) was estimated by 1000 permutations.

### Quantification of GFP-positive cells

A custom Python script (available on request) was employed to segment images based on DAPI (to count the total number of cells) and GFP signal (to quantify the fraction of GFP+ cells).

### Reporting summary

Further information on research design is available in the Nature Research Reporting Summary linked to this article.

## Supplementary information

Supplementary Information

Description of Additional Supplementary Files

Supplementary Data 1

Supplementary Data 2

Supplementary Data 3

Supplementary Data 4

Reporting Summary

## Data Availability

Raw sequencing data were deposited at the Gene Expression Omnibus (GEO) under accession number GSE122743. Processed data for single and clustered circulating tumour cells were obtained from the GEO (GSE41245 and GSE51827, respectively). Bulk RNA-seq profiles for luminal breast cancer samples were downloaded from the GDC (Genomic Data Commons)^40^ data portal (http://portal.gdc.cancer.gov/; July 25, 2018). All the other data supporting the findings of this study are available within the article and its supplementary information files and from the corresponding authors upon reasonable request. A reporting summary for this article is available as a Supplementary Information file.

## Code availability

The R scripts to reproduce the analyses and plots reported in this paper are available from the corresponding authors upon request.
