# Supplementary material for: Single-cell transcriptomics reveals multi-step adaptations to endocrine therapy
Source: Nat Commun. 2019 Sep 2;10:3840. doi: 10.1038/s41467-019-11721-9 (PMC6718416; doi:10.1038/s41467-019-11721-9)
Supplement: Supplementary file 1 — Supplementary Information [file 41467_2019_11721_MOESM1_ESM.pdf]

Single-cell Transcriptomics reveals multi-step adaptations to endocrine therapy

Hong et al.

Supplementary information

Stage	Treatment-naïve HR+/HER2- primary tumors		
	Total Cases	Mutated ESR1	Mutated ESR1 (Fraction)
ALL	655	9	0.014
IA	271	4	0.015
IB	15	0	0.000
IIA	119	1	0.008
IIB	73	3	0.041
IIIA	45	0	0.000
IIIB	6	0	0.000
IIIC	28	0	0.000
IV	98	1	0.010

Supplementary Table 1. Results of the re-analysis of treatment-naïve HR+/HER2- primary tumours from Razavi et al. 2018.

Dataset	Unfiltered Cells	Filtered Cells	Batch
MCF7-LTED	2,325	1,944	A
MCF7	1,310	1,215	A
MCF7 CD44H +E2	8,750	4,981	B
MCF7 CD44H -E2 2days	18,261	4,999	B
MCF7 CD44H -E2 4days	1,726	1,649	C
MCF7 CD44H -E2 7days	2,318	2,129	C
MCF7 CD44L +E2	16,070	4,998	B
MCF7 CD44L -E2 2days	5,178	4,977	B
MCF7 CD44L -E2 4days	2,139	2,013	C
MCF7 CD44L -E2 7days	2,893	2,697	C
T47D-LTED	4,567	4,272	D
T47D	4,768	4,233	D
T47D CD44H +E2	3,392	3,280	D
T47D CD44H -E2 2days	4,120	4,020	D

Supplementary Table 2. Summary statistics for all scRNA-seq samples reported in this study. For each sample, the total number of captured cells (unfiltered) and high-quality cells (filtered) are indicated, along with the batch.

No. nodes in common between 3 largest communities in the merged network from Figure 3 and the re-run merged network:

	Community 1	Community 2	Community 3
**Community 1**	171	0	3
**Community 2**	0	62	0
**Community 3**	0	0	109

No. nodes in common between 3 largest communities in the re-run merged network and the CD44high network:

	Community 1	Community 2	Community 3
**Community 1**	65	0	3
**Community 2**	0	53	1
**Community 3**	1	0	84

No. nodes in common between 3 largest communities in the re-run merged network and the CD44low network:

	Community 1	Community 2	Community 3
**Community 1**	105	0	4
**Community 2**	0	57	0
**Community 3**	0	1	88

Supplementary Table 3. Consistency of the three main communities identified using (top to bottom) a different initialization of the label propagation algorithm, the network derived from the CD44^{high} data only, the network computed the from CD44^{low} data alone.

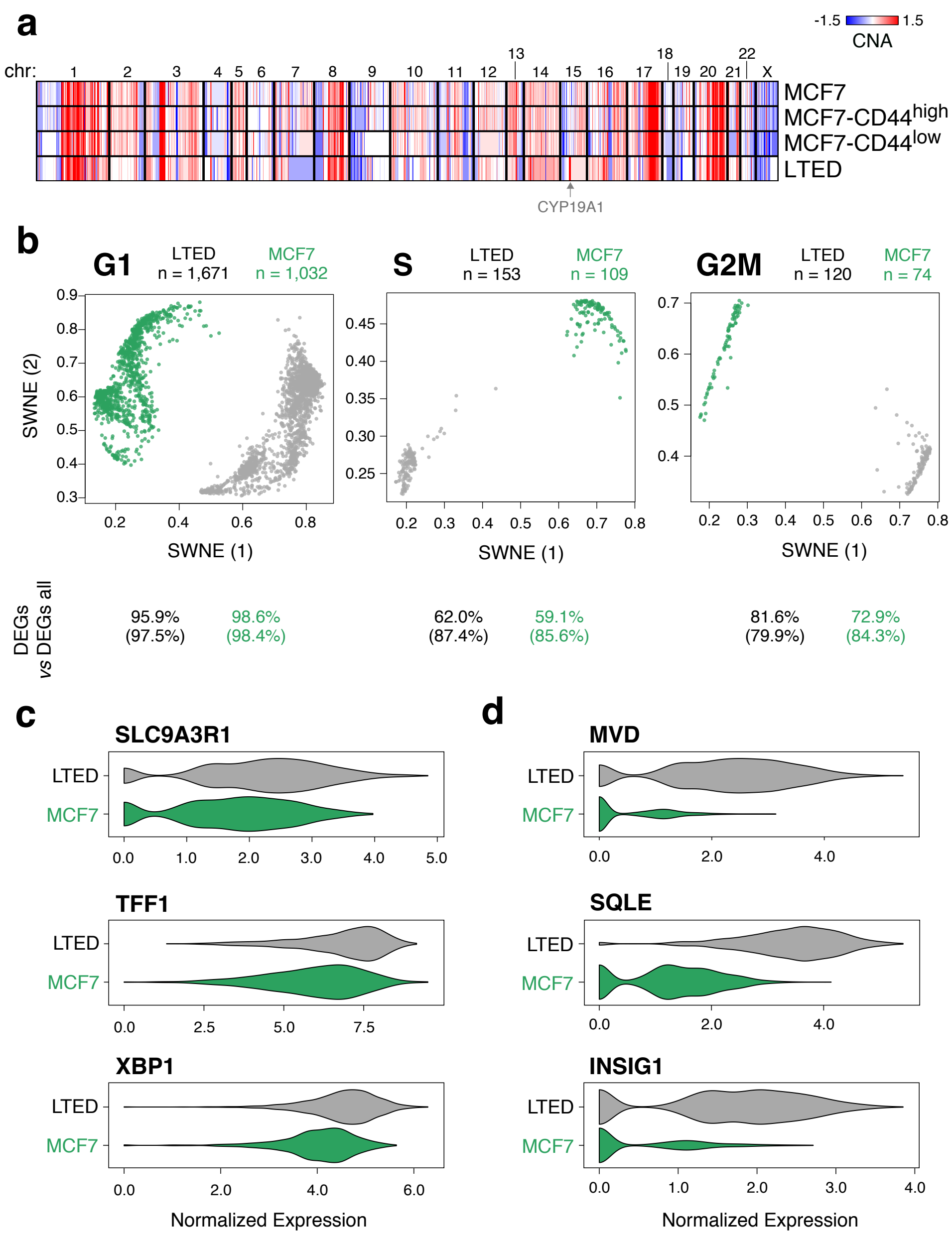

Supplementary Figure 1. (a) Copy number profiles of the MCF7 (full population), MCF7-CD44^{high}, MCF7-CD44^{low} and LTED, as estimated from ChIP-input DNAs. (b-c) Normalized expression of a selection of ER-target genes (b) and genes involved in cholesterol biosynthesis and homeostasis (c) in single MCF7 and LTED cells. (d) Bi-dimensional representation of 3,159 single cell transcriptomes (1,125 MCF7 and 1,944 LTED) (SWNE; *k* = 16) separately for cells inferred to be in different phases of the cell cycle, according to Cyclone. The overlaps of the differentially expressed genes (DEGs) between LTED and MCF7 cells in each cell cycle phase and the DEGs estimated from the entire dataset are shown at the bottom of each plot (black indicates the results for the set of genes showing higher expression in LTED, green those of genes with higher expression in LTED).

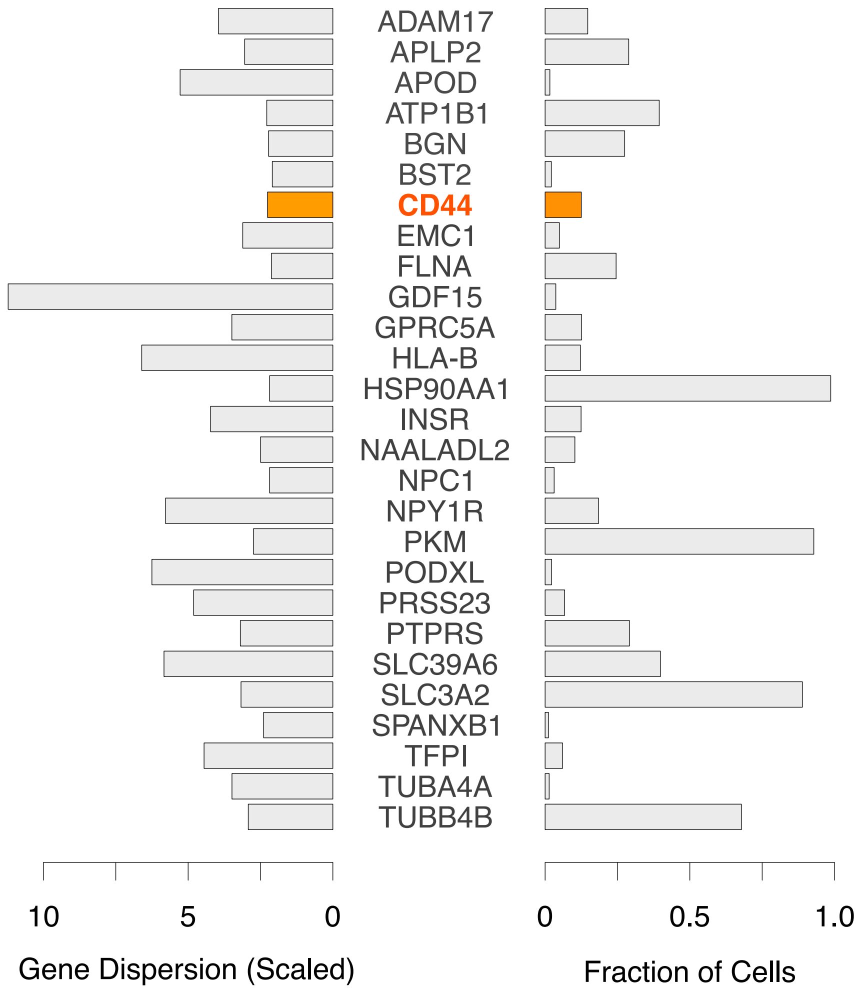

Supplementary Figure 2. Heterogeneously expressed surface markers as estimated from scRNA-seq profiling. Genes were selected as showing dispersion >= 2 (n = 27) out of Fraction of Cells >= 1% (N = 778).

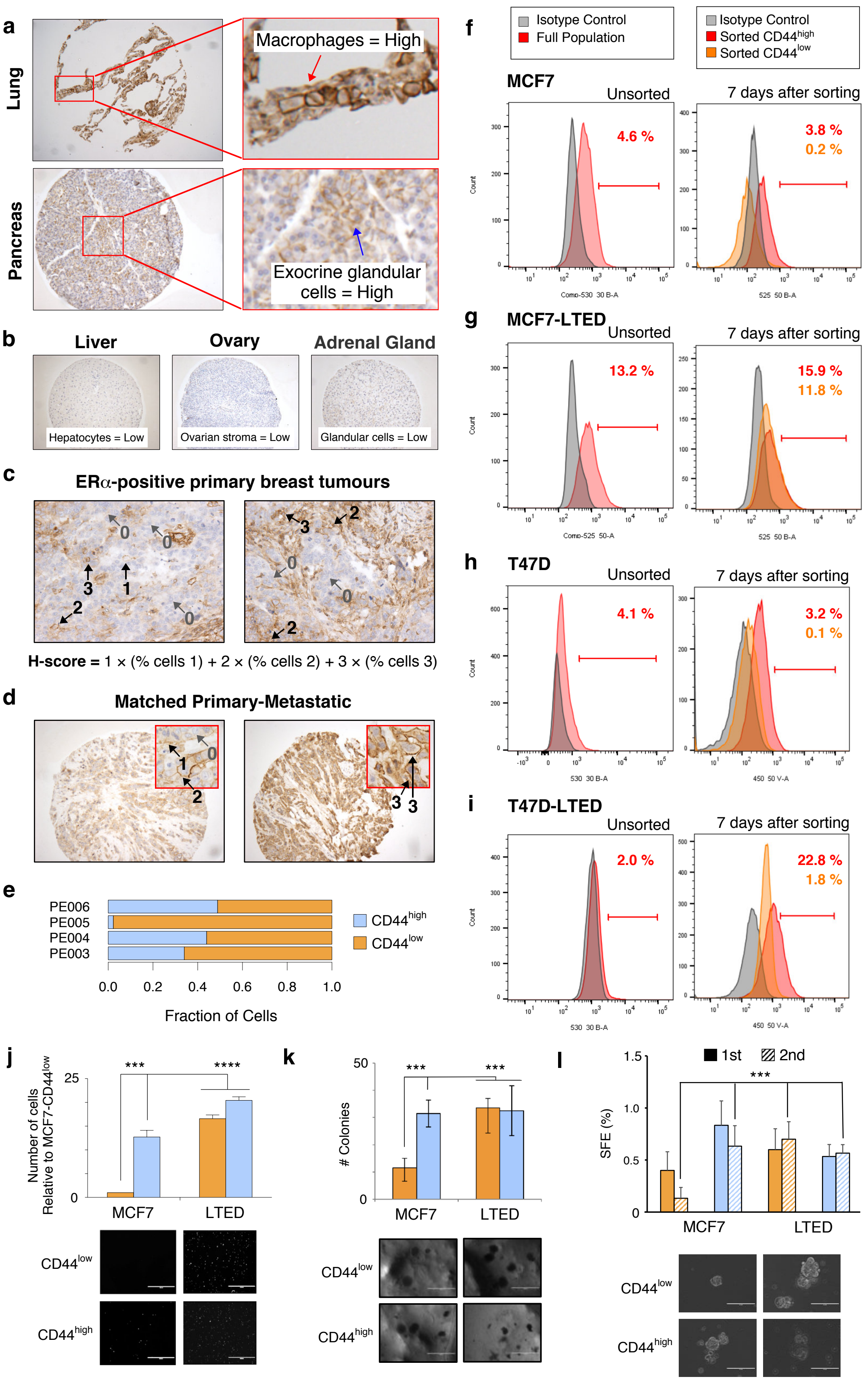

Supplementary Figure 3. (a-d) Immunohistochemical staining of CD44 in two primary tumours (c) and in one matched primary-metastatic pair. Staining from lung and

pancreas (a) and from liver, ovary and adrenal gland (b) are shown as positive and negative controls, respectively. (e) FACS quantification of $CD44^{high}$ cells in pleural effusion cells from four patients. (f-i) FACS quantification of $CD44^{high}$ cells in MCF7 (f), MCF7-LTED (g), T47D (h) or T47D-LTED (i) cells. Unsorted populations (left) were compared to sorted $CD44^{high}$ or $CD44^{low}$ cells cultured for 7 days in full medium (right). (j-k) Invasion (h) and colony formation (i) assays for $CD44^{high}$ and $CD44^{low}$ cells sorted from either MCF7 or LTED cells. Representative images shown below the plots (*p*-values estimated using two-tailed paired *t*-tests). (l) Mammosphere-forming efficiency (SFE) of for $CD44^{high}$ and $CD44^{low}$ cells sorted from either MCF7 or LTED cells. Representative images shown below the plots (*p*-values through ANOVA). Scale bars = 1,000 μm (j), 100 μm (k), 200 μm (l). Standard deviation of the mean estimated from 3 replicates is shown. * $p <= 0.05$, ** $p <= 0.01$, *** $p <= 0.001$, **** $p <= 0.0001$.

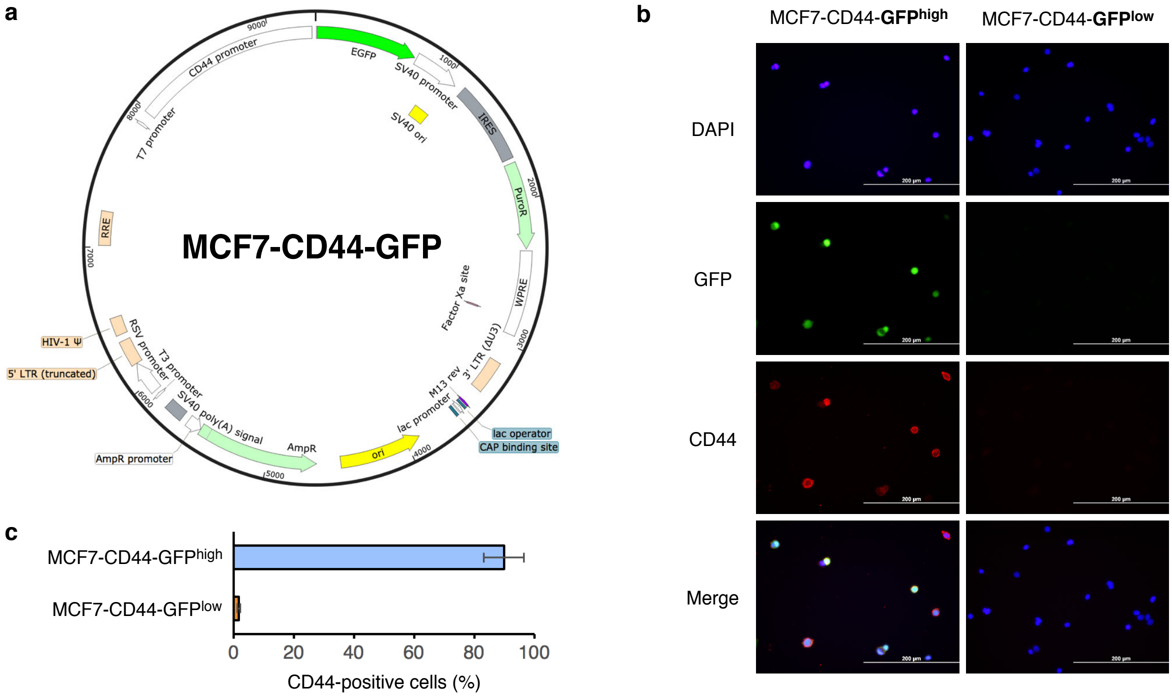

Supplementary Figure 4. (a) Overview of the construct used to derive MCF7 stably expressing GFP under the control of the promoter of the CD44 gene. (b) Representative immunofluorescent images of sorted GFP^{high} and GFP^{low} cells, stained for DAPI and CD44. Scale bar = 200 μm. (c) Quantification of the fraction of sorted GFP^{high} and GFP^{low} cells from (b). Standard deviation of the mean estimated from 3 replicates is shown.

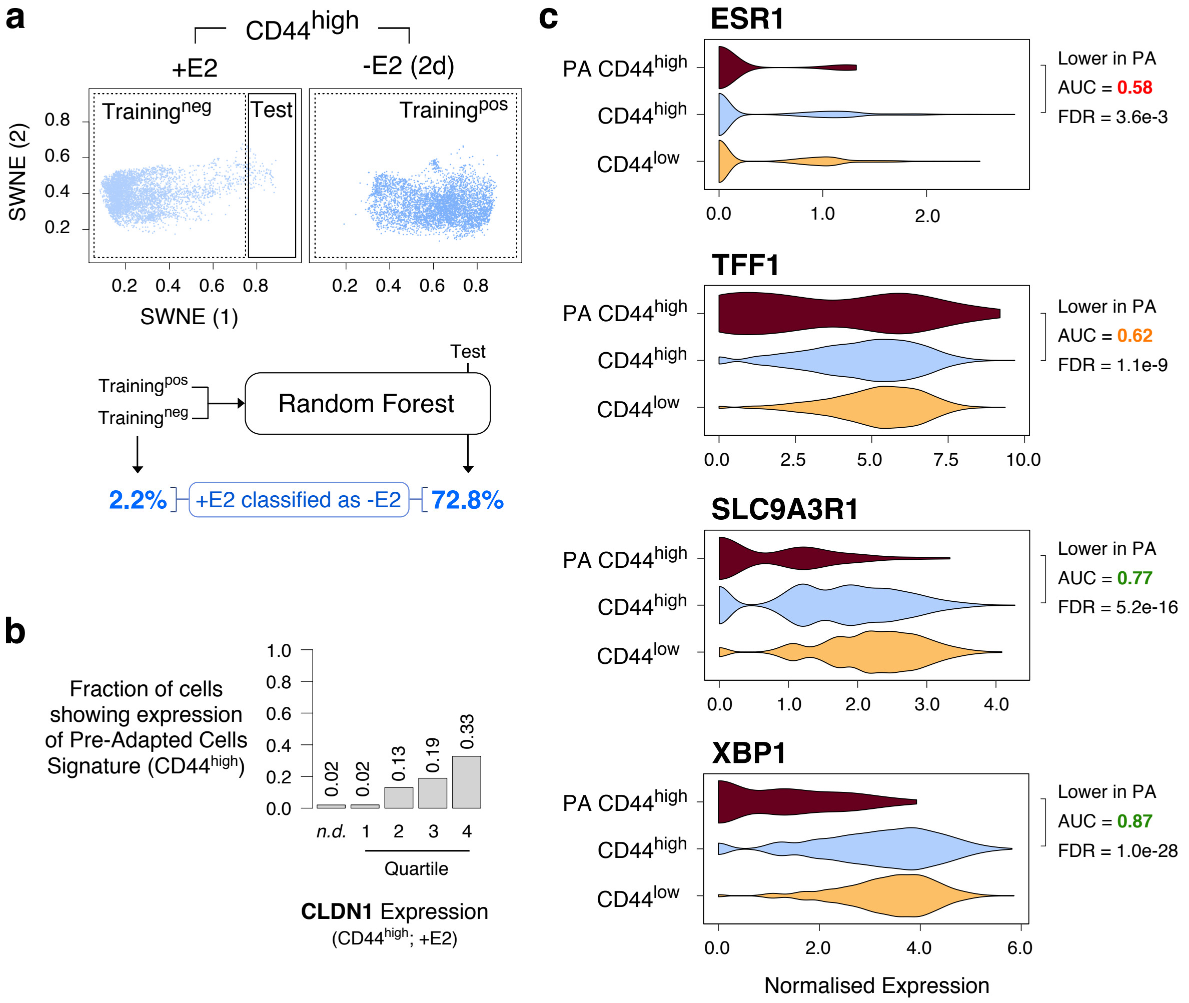

Supplementary Figure 5. (a) A random forest classifier was trained to discriminate starved (-E2) CD44^{high} cells (dashed box; right) from those in treatment-naïve (+E2) condition that were not showing features of pre-adapted (PA) cells (dashed box; left). The trained model was then used to classify PA cells (solid box; left). (b) AUCell quantification of the fraction of CD44^{high} single-cells (+E2) showing a transcriptomic profile compatible with the PA signature, after stratification for the expression level of CLDN1. (c) Normalized expression of ESR1 and of selected ER-target genes in the indicated subpopulations of MCF7 cells.

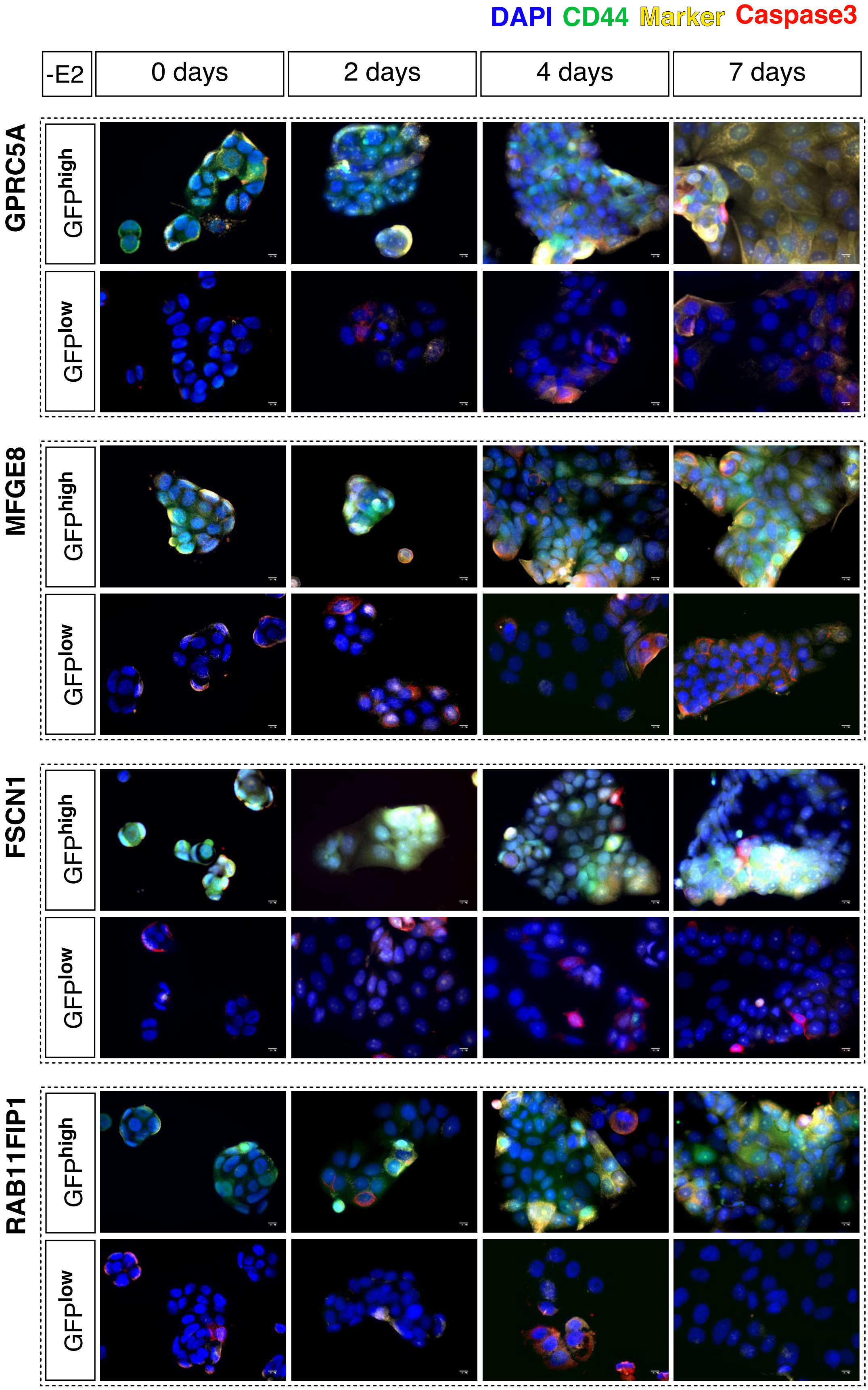
DAPI CD44 Marker Caspase3
-E2
0 days
2 days
4 days
7 days
GPRC5A
GFPhigh
GFPlow
MFGE8
GFPhigh
GFPlow
FSCN1
GFPhigh
GFPlow
RAB11FIP1
GFPhigh
GFPlow

Supplementary Figure 6. Representative images of sorted CD44^{high} or CD44^{low} cells, stained for DAPI, CD44, Caspase3 and the indicated marker gene at different time points of oestradiol starvation. Scale bar = 10 μm.

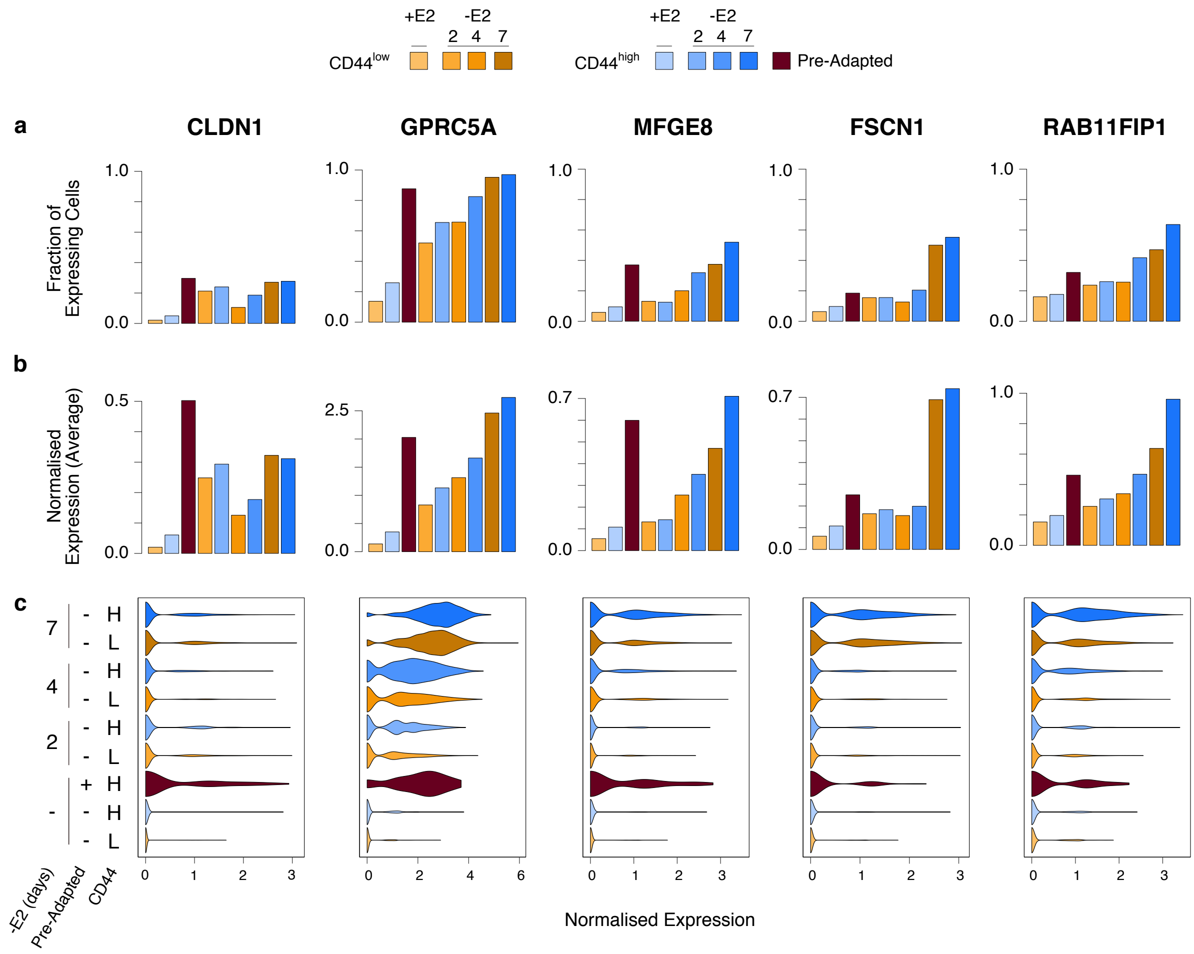

Supplementary Figure 7. (a-c) For the indicated genes, the fraction of cells expressing them (a) along with the average (b) and the distribution (c) of normalized expression values are shown for the indicated subpopulations (top panel).

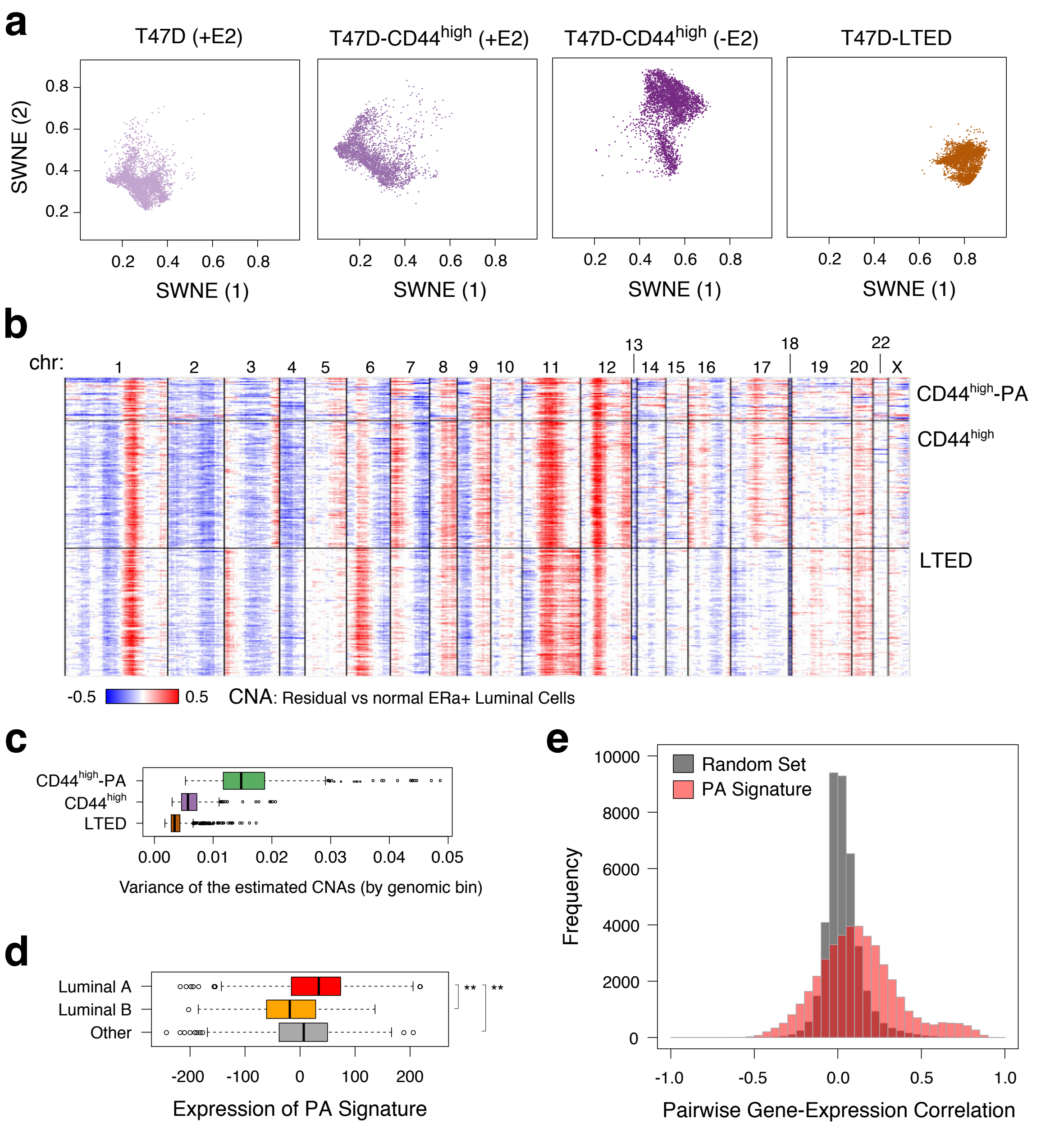

Supplementary Figure 8. (a) Bi-dimensional representation of 15,805 transcriptomes from single T47D cells, split by capture (Supplementary Table 2) (SWNE; *k* = 20). (b) Copy number profiles (estimated from scRNA-seq profiles) of pre-adapted (PA) cells (n = 46), along with the four times this number of LTED and $CD44^{high}$ (not PA) cells. (c) Box plot showing the distribution of the variance in the estimated copy number alterations across the cells, per group (each data point is the variance across cells, given a genomic bin). (d) Expression of the PA signature in bulk RNA-seq samples from TCGA (** $p <= 2.2e-16$; Wilcoxon rank-sum test). (e) Distributions of Spearman's rank correlation coefficients between expression profiles of genes in the PA signature (red) or a set of randomly picked genes of the same size (grey), across bulk RNA-seq samples from TCGA classified as luminal A. Box plots show median, interquartile values, range, and outliers (individual points).
