# Supplementary material for: Single-cell transcriptomics reveals multi-step adaptations to endocrine therapy
Source: Nat Commun. 2019 Sep 2;10:3840. doi: 10.1038/s41467-019-11721-9 (PMC6718416; doi:10.1038/s41467-019-11721-9)
Supplement: Supplementary file 2 — Description of Additional Supplementary Files [file 41467_2019_11721_MOESM2_ESM.pdf]

Description of Additional Supplementary Files

File Name: Supplementary Data 1
Description: Differentially expressed genes (DEGs) between MCF7-CD44^{high} (CD44H), MCF7-CD44^{low} (CD44L) and LTED. For each comparison, the false discovery rate (FDR) estimated using MAST, along with the area under the curve (AUC) and the fraction of cells expressing each DEG in the compared subpopulations are indicated.

File Name: Supplementary Data 2
Description: Network reconstructed using PIDC. For each inferred regulatory interaction, the two nodes are shown (genes 1 and 2), along with information indicating whether the edge is supported by either the network inferred from CD44^{high} and CD44^{low} data only, or both. For both genes, the table indicates the network component, whether the gene falls into one of the three main network communities (i.e. the three larger components) and into any of the two sub-communities of community 1, and whether the gene is part of the PA signature.

File Name: Supplementary Data 3
Description: Genes either up- or down-regulated in pre-adapted (PA) cells, using either the SWNE-based or the Random-Forest-based (RF) approach. For each list, the false discovery rate (FDR) estimated using MAST, along with the area under the curve (AUC) and the fraction of cells expressing each DEG in the compared subpopulations are indicated.

File Name: Supplementary Data 4
Description: Genes either up- or down-regulated in T47D pre-adapted (PA) cells. For each list, the false discovery rate (FDR) estimated using MAST, along with the area under the curve (AUC) and the fraction of cells expressing each DEG in the compared subpopulations are indicated.
