# Supplementary material for: Single-cell transcriptomics reveals multi-step adaptations to endocrine therapy
Source: Nat Commun. 2019 Sep 2;10:3840. doi: 10.1038/s41467-019-11721-9 (PMC6718416; doi:10.1038/s41467-019-11721-9)
Supplement: Supplementary file 7 — Reporting Summary [file 41467_2019_11721_MOESM7_ESM.pdf]

nature research

Corresponding author(s): Luca Magnani

Last updated by author(s): Jul 24, 2019

Reporting Summary

Nature Research wishes to improve the reproducibility of the work that we publish. This form provides structure for consistency and transparency in reporting. For further information on Nature Research policies, see Authors & Referees and the Editorial Policy Checklist.

Statistics

For all statistical analyses, confirm that the following items are present in the figure legend, table legend, main text, or Methods section.

n/a	Confirmed	
☐	☒	The exact sample size (*n*) for each experimental group/condition, given as a discrete number and unit of measurement
☐	☒	A statement on whether measurements were taken from distinct samples or whether the same sample was measured repeatedly
☐	☒	The statistical test(s) used AND whether they are one- or two-sided<br>*Only common tests should be described solely by name; describe more complex techniques in the Methods section.*
☐	☒	A description of all covariates tested
☐	☒	A description of any assumptions or corrections, such as tests of normality and adjustment for multiple comparisons
☐	☒	A full description of the statistical parameters including central tendency (e.g. means) or other basic estimates (e.g. regression coefficient) AND variation (e.g. standard deviation) or associated estimates of uncertainty (e.g. confidence intervals)
☐	☒	For null hypothesis testing, the test statistic (e.g. *F*, *t*, *r*) with confidence intervals, effect sizes, degrees of freedom and *P* value noted<br>*Give P values as exact values whenever suitable.*
☒	☐	For Bayesian analysis, information on the choice of priors and Markov chain Monte Carlo settings
☒	☐	For hierarchical and complex designs, identification of the appropriate level for tests and full reporting of outcomes
☒	☐	Estimates of effect sizes (e.g. Cohen's *d*, Pearson's *r*), indicating how they were calculated

Our web collection on statistics for biologists contains articles on many of the points above.

Software and code

Policy information about availability of computer code

Data collection	*Provide a description of all commercial, open source and custom code used to collect the data in this study, specifying the version used OR state that no software was used.*
Data analysis	R v3; cellRanger v2.1.1; R packages (cellranger v1.1.0; Seurat v2.3.4; Scran v1.6.9; biomaRt v2.34.2; zoo v1.8-3; dendextend v1.8.0; MAST v1.4.1; ROCR; AUCell v1.0.0; class v7.3-14); bowtie2 v2.3.4.3; samtools v1.9; CNVkit v.0.9.4.dev0; Picard v2.1.1; PIDC; custom R and python scripts

For manuscripts utilizing custom algorithms or software that are central to the research but not yet described in published literature, software must be made available to editors/reviewers. We strongly encourage code deposition in a community repository (e.g. GitHub). See the Nature Research guidelines for submitting code & software for further information.

Data

Policy information about availability of data

All manuscripts must include a data availability statement. This statement should provide the following information, where applicable:

- Accession codes, unique identifiers, or web links for publicly available datasets
- A list of figures that have associated raw data
- A description of any restrictions on data availability

Raw sequencing data was deposited at the Gene Expression Omnibus (GEO) under accession number GSE122743. Processed data for single and clustered circulating tumor cells were obtained from the GEO (GSE41245 and GSE51827, respectively). Bulk RNA-seq profiles for luminal breast cancer samples were downloaded from the GDC (Genomic Data Commons) data portal (http://portal.gdc.cancer.gov/; July 25, 2018). All the other data supporting the findings of this study are available within the article and its supplementary information files and from the corresponding authors upon reasonable request. A reporting summary for this article is available as a Supplementary Information file.

Field-specific reporting

Please select the one below that is the best fit for your research. If you are not sure, read the appropriate sections before making your selection.

[x] Life sciences [ ] Behavioural & social sciences [ ] Ecological, evolutionary & environmental sciences

For a reference copy of the document with all sections, see nature.com/documents/nr-reporting-summary-flat.pdf

Life sciences study design

All studies must disclose on these points even when the disclosure is negative.

Sample size	No sample size calculations were performed.
Data exclusions	No data were excluded from the analyses.
Replication	All attempts at replication were successful.
Randomization	No randomization was performed.
Blinding	No blinding was performed.

Reporting for specific materials, systems and methods

We require information from authors about some types of materials, experimental systems and methods used in many studies. Here, indicate whether each material, system or method listed is relevant to your study. If you are not sure if a list item applies to your research, read the appropriate section before selecting a response.

Materials & experimental systems

n/a	Involved in the study	
[ ]	[x]	Antibodies
[ ]	[x]	Eukaryotic cell lines
[x]	[ ]	Palaeontology
[x]	[ ]	Animals and other organisms
[ ]	[x]	Human research participants
[ ]	[x]	Clinical data

Methods

n/a	Involved in the study	
[x]	[ ]	ChIP-seq
[ ]	[x]	Flow cytometry
[x]	[ ]	MRI-based neuroimaging

Antibodies

Antibodies used	Anti-ERa antibody (Vector Laboratories, VP-E613) 1:100 for immunofluorescence (IF) and anti-ER[illegible] (Santa Cruz, HC-20) 1:1000 for western blot (WB), anti-CD44 antibody (Santa Cruz, sc-7297,) 1:200 for IF and 1:100 for immunohistochemistry (IHC), anti-pan Cytochemistry antibody (Abcam, ab17154) 1:200 for IF, anti-FGFR4 antibody (Abcam, ab44971) 1:100 for IF, anti-FSCN1(Sigma, HPA005723) 1:100 for IF, anti-MFGE8 (Sigma, HPA002807) 1:100 for IF, anti-RAB11FIP1 (Sigma, HPA023904) 1:100 for IF, anti-GPRC5A (Sigma, HPA007928) 1:100 for IF, anti-caspase3 (Merk, MAB10753) 1:100 for IF.
Validation	The manufacturers provide the validation documents of the antibodies. When we performed the experiments, we included positive controls and negative controls.

Eukaryotic cell lines

Policy information about cell lines

Cell line source(s)	MCF7 and long-term oestrogen-deprived cells (LTED) were kindly provided by Philippa Darbre and T47D and LTED were kindly provided by Matthew Ellis.
Authentication	None of the cell lines used were authenticated.
Mycoplasma contamination	All the cell lines tested negative for mycoplasm contamination.
Commonly misidentified lines (See ICLAC register)	*Name any commonly misidentified cell lines used in the study and provide a rationale for their use.*

Human research participants

Policy information about studies involving human research participants

Population characteristics	Female estrogen receptor alpha positive breast cancer patients with 50-70 year old were included in the study.
Recruitment	The patients were recruited into the tissue bank according to the institutional protocol.
Ethics oversight	Imperial College Healthcare NHS Trust Tissue Bank and the European Institute of Oncology Tissue Bank approved the study.

Note that full information on the approval of the study protocol must also be provided in the manuscript.

Clinical data

Policy information about clinical studies

All manuscripts should comply with the ICMJE guidelines for publication of clinical research and a completed CONSORT checklist must be included with all submissions.

Clinical trial registration	*Provide the trial registration number from ClinicalTrials.gov or an equivalent agency.*
Study protocol	*Note where the full trial protocol can be accessed OR if not available, explain why.*
Data collection	*Describe the settings and locales of data collection, noting the time periods of recruitment and data collection.*
Outcomes	*Describe how you pre-defined primary and secondary outcome measures and how you assessed these measures.*

Flow Cytometry

Plots

Confirm that:

☒ The axis labels state the marker and fluorochrome used (e.g. CD4-FITC).

☒ The axis scales are clearly visible. Include numbers along axes only for bottom left plot of group (a 'group' is an analysis of identical markers).

☒ All plots are contour plots with outliers or pseudocolor plots.

☒ A numerical value for number of cells or percentage (with statistics) is provided.

Methodology

Sample preparation	Cells were cultured to 70 to 80% confluence and detached from the cell culture flasks using EDTA. Cell pellets were obtained and washed with cold phosphate-buffered saline (PBS) containing 1% FCS and 5mM EDTA. All further steps were performed on ice and all centrifugation steps at 4°C.
Instrument	We used FACSAria and Aria III (BD Biosciences) in the LMS/NIHR Flow Cytometry Facility at Imperial.
Software	We used FlowJo software to analyze the data.
Cell population abundance	We sorted cells according to the level of GFP expression at 50,000 cells for scRNA seq. We checked the purity after sorting and they showed around 98%.
Gating strategy	Gating was set to relevant isotype control (IgG-FITC)-labeled cells or unstained cells for each cell line. FSC and SSC were set to remove cell debris and doublets. Propidium iodide (Bio-Rad, 1351101) and DRAQ7 (BioLegend, 424001) were used for the dead cell removal.

☒ Tick this box to confirm that a figure exemplifying the gating strategy is provided in the Supplementary Information.
